# Commented checklist of European Gelechiidae (Lepidoptera)

**DOI:** 10.3897/zookeys.921.49197

**Published:** 2020-03-24

**Authors:** Peter Huemer, Ole Karsholt

**Affiliations:** 1 Naturwissenschaftliche Sammlungen, Tiroler Landesmuseen Betriebsges.m.b.H., Innsbruck, Austria Naturwissenschaftliche Sammlungen Innsbruck Austria; 2 Zoological Museum, Natural History Museum of Denmark, Universitetsparken 15, DK-2100 Copenhagen, Denmark Zoological Museum, Natural History Museum of Denmark Co­penhagen Denmark

**Keywords:** Europe, species diversity, cryptic diversity, DNA barcoding, synonymy, new combination

## Abstract

The checklist of European Gelechiidae covers 865 species, belonging to 109 genera, with three species records which require confirmation. Further, it is the first checklist to include a complete coverage of proved synonyms of species and at generic level. The following taxonomic changes are introduced: *Pseudosophronia constanti* (Nel, 1998) **syn. nov.** of *Pseudosophronia exustellus* (Zeller, 1847), *Metzneria expositoi* Vives, 2001 **syn. nov.** of *Metzneria aestivella* (Zeller, 1839); *Sophronia ascalis* Gozmány, 1951 **syn. nov.** of *Sophronia grandii* Hering, 1933, *Aproaerema incognitana* (Gozmány, 1957) **comb. nov.**, *Aproaerema cinctelloides* (Nel & Varenne, 2012) **comb. nov.**, *Aproaerema azosterella* (Herrich-Schäffer, 1854) **comb. nov.**, *Aproaerema montanata* (Gozmány, 1957) **comb. nov.**, *Aproaerema cincticulella* (Bruand, 1851) **comb. nov.**, *Aproaerema buvati* (Nel, 1995) **comb. nov.**, *Aproaerema linella* (Chrétien, 1904) **comb. nov.**, *Aproaerema captivella* (Herrich-Schäffer, 1854) **comb. nov.**, *Aproaerema semicostella* (Staudinger, 1871) **comb. nov.**, *Aproaerema steppicola* (Junnilainen, 2010) **comb. nov.**, *Aproaerema cottienella* (Nel, 2012) **comb. nov.**, *Ptocheuusa cinerella* (Chrétien, 1908) **comb. nov.**, *Pragmatodes melagonella* (Constant, 1895) **comb. nov.**, *Pragmatodes albagonella* (Varenne & Nel, 2010) **comb. nov.**, *Pragmatodes parvulata* (Gozmány, 1953) **comb. nov.**, *Oxypteryx nigromaculella* (Millière, 1872) **comb. nov.**, *Oxypteryx wilkella* (Linnaeus, 1758) **comb. nov.**, *Oxypteryx ochricapilla* (Rebel, 1903) **comb. nov.**, *Oxypteryx superbella* (Zeller, 1839) **comb. nov.**, *Oxypteryx mirusella* (Huemer & Karsholt, 2013) **comb. nov.**, *Oxypteryx baldizzonei* (Karsholt & Huemer, 2013) **comb. nov.**, *Oxypteryx occidentella* (Huemer & Karsholt, 2011) **comb. nov.**, *Oxypteryx libertinella* (Zeller, 1872) **comb. nov.**, *Oxypteryx gemerensis* (Elsner, 2013) **comb. nov.**, *Oxypteryx deserta* (Piskunov, 1990) **comb. nov.**, *Oxypteryx unicolorella* (Duponchel, 1843) **comb. nov.**, *Oxypteryx nigritella* (Zeller, 1847) **comb. nov.**, *Oxypteryx plumbella* (Heinemann, 1870) **comb. nov.**, *Oxypteryx isostacta* (Meyrick, 1926) **comb. nov.**, *Oxypteryx helotella* (Staudinger, 1859) **comb. nov.**, *Oxypteryx parahelotella* (Nel, 1995) **comb. nov.**, *Oxypteryx graecatella* (Šumpich & Skyva, 2012) **comb. nov.**; *Aproaerema genistae* (Walsingham, 1908) **comb. rev.**, *Aproaerema thaumalea* (Walsingham, 1905) **comb. rev.**; *Dichomeris neatodes* Meyrick, 1923 **sp. rev.**; *Caryocolum horoscopa* (Meyrick, 1926) **stat. rev.**; *Ivanauskiella occitanica* (Nel & Varenne, 2013) **sp. rev.**; *Apodia martinii* Petry, 1911 **sp. rev.**; *Caulastrocecis cryptoxena* (Gozmány, 1952) **sp. rev.** Following Article 23.9.2 ICZN we propose *Caryocolum blandella* (Douglas, 1852) (*Gelechia*) **nom. protectum** and *Caryocolum signatella* (Eversmann, 1844) (*Lita*) **nom. oblitum.**

## Introduction

Lepidoptera, butterflies and moths, are among the best-known insects, and due to a long tradition of studying Lepidoptera in Europe our knowledge of European Lepidoptera is more comprehensive compared to other parts of the world. Even though Lepidoptera is a well-defined group they exhibit a huge diversity in size, colour and wing markings. Whereas everybody can recognize a butterfly the vast majority of Lepidoptera are small and often dull coloured insects. One such group is the family Gelechiidae. They have for a long time been rather neglected by most lepidopterists mainly due to their external similarity and lack of resources for their identification. Over the last couple of decades, the latter problem has partly been addressed, e.g., Elsner et al. (1999), Huemer and Karsholt (1999, 2010), and at the same time there has been an increasing research interest in the Gelechiidae, resulting in a number of smaller and larger taxonomic reviews and faunistic publications (see reference list) dealing with these moths. However, what was becoming increasingly a hindrance for ongoing research was the lack of an updated checklist of European Gelechiidae. In particular, when planning an extensive DNA barcoding project for the family (Huemer et al. 2020), this deficit became obvious and therefore the authors decided to compile such a checklist for this and future requirements.

A checklist is the most basic taxonomic work on a group of organisms. It can be alphabetical or systematic, viz. trying to reflect the current knowledge of the relationship of the included taxa. This checklist is in systematic order, and it moreover includes synonyms and annotations. Its aim is to present an updated overview of the Gelechiidae known from Europe. This is highly appropriate as nearly a quarter of the currently known species have been described since 1990 (Huemer et al. 2020).

This checklist of European Gelechiidae is the first one to include all known synonyms of genera and species of Europaean Gelechiidae. It is mainly based on data published in Fauna Europaea (Karsholt 2004–2019) but supplemented with numerous published and unpublished additions and corrections from the last few years. It covers all currently accepted species known from the European fauna and their synonyms. Subspecies are not given separate entries, but listed among synonyms, though marked as subspecies. Subgenera are listed among generic synonyms. The considerable number of likely undescribed species (Huemer et al. 2020) are not included in the list.

**Figure 1.**
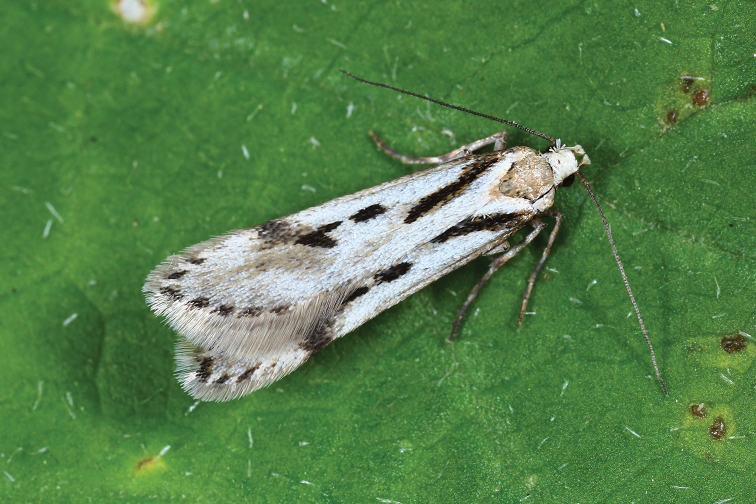
Alpine species of *Sattleria* are a striking example of long underestimated species diversity (photograph Michel Billard).

Taxonomically critical genera and species, especially possible cases of cryptic diversity (Fig. 1) manifested by divergent DNA barcodes, are commented on in detail (see also Huemer et al. 2020).

## Materials and methods

### Geographic restriction

**Figure 2.**
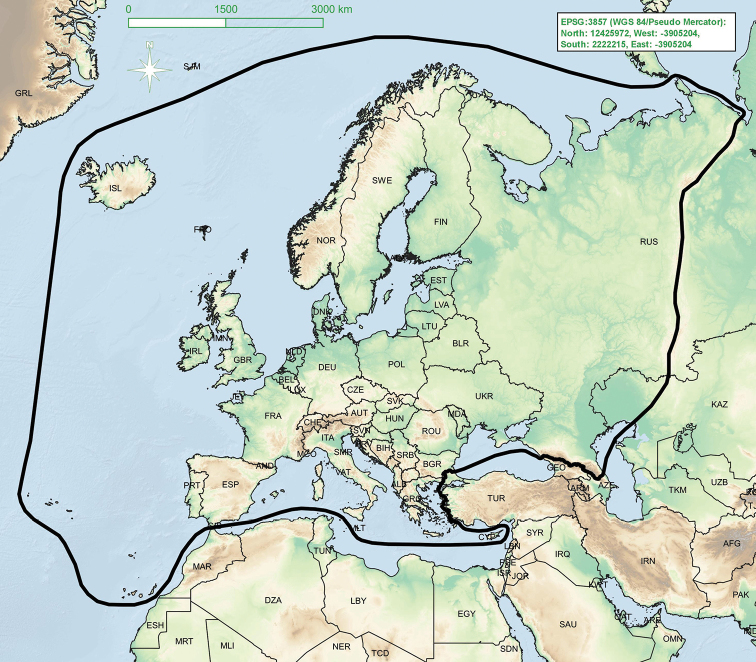
Geographical boundaries of research area.World boundaries: https://www.arcgis.com/; SRTM-Data: https://dds.cr.usgs.gov/srtm/version2_1/SRTM3/.

For the purpose of the present checklist we define Europe in a broad sense, which includes the Ural Mountains, Russian parts of the Caucasus, the ‘European’ part of Kazakhstan, the Mediterranean islands and the Macaronesian Islands (except Cape Verde) (Fig. 2).

The inclusion of the Russian parts of the Caucasus only added four species to the list (*Acompsia caucasella* Huemer & Karsholt, *Neofriseria caucasicella* Sattler, *Chionodes caucasiella* Huemer & Sattler and *Scrobipalpa caucasica* (Povolný)), which is surprising. One would expect a richer gelechiid fauna to occur in this vast and diverse mountain system. However, most likely the species inventory is simply underestimated as only few lepidopterists have done field research in this area so far.

### Content and structure of the checklist

The checklist is restricted to described nominal taxa. Potentially undescribed species (Huemer et al. 2020) are not included. Species introduced from other parts of the World are only included if they are known to have been naturalized within the area described above. Doubtful, though possible, records of occurrence are considered in the checklist and marked with an asterisk *, whereas confirmed incorrect records and doubtful species (*taxa incertae sedis*) are not listed. Names applied to misidentified taxa are listed only in cases where the incorrect taxonomy has been widely used or where the misidentification can easily cause misunderstandings. These are marked with *auct.* (= of authors).

### Systematic arrangement

The higher classification follows the molecular study of Karsholt et al. (2013), whereas the listed order of genera and species is largely according to published revisions and data from Huemer et al. (2020).

### Synonymy

Although our knowledge of European Gelechiidae has increased much over the last years, there are still available species-group names in the family which have not yet been associated with known species. Very few of these are likely to represent additional taxa, whereas most cases will be synonyms. Furthermore, several of the published synonyms need taxonomic re-assessment. We have not made special efforts to search for type specimens of such taxa for the purpose of the present checklist, and they should be considered in connection with taxonomic revisions within the Gelechiidae.

### Gender agreement

Many species-group names of European Gelechiidae have been combined in different genera since they were first made available. Following article 31.2 of the International Code of Zoological Nomenclature (ICZN 1999) these names require gender agreement between specific and generic names. However, we follow the widely accepted proposals by Sommerer (2002) in Lepidoptera and keep the original spelling of species names to avoid unnecessary instability (van Nieukerken et al. 2019).

### Molecular species delimitation

DNA barcodes have been sequenced for a significant number of the species included in the inventory (741 nominal species with sequences > 500 bp). These supported the compilation of the checklist and helped identify and fix yet unpublished synonyms and the systematic position of some species. Details to species and specimens are available on BOLD (Ratnasingham 2018) in the public dataset “Lepidoptera (Gelechiidae) of Europe” under the DOI: https://doi.org/10.5883/DS-GELECHEU (see also Huemer et al 2020).

We tested the congruence of morphologically based species determinations and COI sequence data with the Barcode Index Number (BIN), a methodology recently proposed by Ratnasingham and Hebert (2013). This system clusters sequences into Operational Taxonomic Units (OTUs) regardless of their previous taxonomic assignment. It is based on a two-stage algorithm that groups the sequences in a cluster and automatically assigns new sequences. All high-quality sequences > 500 bp are recorded independently of the project origin and assigned to a BIN. Though BINs reflect classical Linnean taxonomy to a high level they were not used uncontested (Huemer et al 2020). We found 114 morphologically delimited species with multiple BINs that are potential cases of cryptic diversity, particularly cases with BIN distances > 3%, and these are therefore discussed in the comments. However, there is clear evidence that no species delimiting threshold values exist in Lepidoptera (Kekkonen et al. 2015) and therefore all cases of barcode divergence require further and integrative analysis in the future. Such work was largely outside the scope of this paper which principally followed current taxonomy and only exceptionally considered obvious taxonomic issues. An in-depth taxonomical analysis will also be necessary for 65 clusters with a unique BIN which remained unidentified to species level from morphology and which are not considered in the checklist itself, and for 55 cases of BIN-sharing (see also Huemer et al. (2020)).

## Results

### Overview

The checklist covers 865 nominal species of European Gelechiidae belonging to 109 genera, including 3 species with doubtful records (*). The majority belong to Gelechiinae (445 spp.), followed by Anomologinae (253 spp.), Anacampsinae (89 spp.), Dichomeridinae (47 spp.), Apatetrinae (29 spp.), and Thiotrichinae (5 spp.) (Table 1).

**Table 1.** Number of described species per tribe/subfamily.

Higher taxa	Species no.
**Gelechiidae Stainton, 1854**	**865**
**Anacampsinae Bruand d’Uzelle, 1851**	**89**
Anacampsini Bruand d’Uzelle, 1851	67
Chelariini Le Marchand, 1947	22
**Dichomeridinae Hampson, 1918**	**47**
**Apatetrinae Le Marchand, 1947**	**29**
Pexicopiini Hodges, 1986	6
Apatetrini Le Marchand, 1947	23
**Thiotrichinae Karsholt, Mutanen, Lee & Kaila, 2013**	**5**
**Anomologinae Meyrick, 1926**	**253**
**Gelechiinae Stainton, 1854**	**445**
Gelechiini Stainton, 1854	132
Gnorimoschemini Povolný, 1964	240
Litini Bruand d’Uzelle 1859	73

### Taxon excluded from the Gelechiidae

A single species originally described in the Gelechiidae is excluded from the family, viz. *Brachmia infuscatella* Rebel, 1940, and is transferred to Autostichidae without generic assignation.

### Checklist

Numbers [1] – [202] refer to comments; * refers to doubtful records for the European fauna.

**Gelechiidae Stainton, 1854**

**Anacampsinae Bruand d’Uzelle, 1851 [1**]

Stomopteryginae Heslop, 1938, unavailable

**Anacampsini Bruand d’Uzelle, 1851**

***Stomopteryx* Heinemann, 1870 [2**]

*Inotica* Meyrick, 1913

*Acraeologa* Meyrick, 1921

*Kahelia* Turati, 1922, unavailable

*Stomopteryx detersella* (Zeller, 1847)

*egenella* (Herrich-Schäffer, 1851), unavailable

*palermitella* (La Harpe, 1860)

*tenuisignella* Turati, 1924

*obliterella* Turati, 1924, unavailable

*Stomopteryx bolschewickiella* (Caradja, 1920)

*Stomopteryx nugatricella* Rebel, 1893 [**3**]

*Stomopteryx mongolica* Piskunov, 1975 [**3**]

*Stomopteryx lineolella* (Eversmann, 1844) [**3**]

*Stomopteryx basalis* (Staudinger, 1876)

*oxychalca* (Meyrick, 1937)

*Stomopteryx deverrae* (Walsingham, 1905) [**4**]

*Stomopteryx flavoclavella* Zerny, 1935 [**5**]

*Stomopteryx remissella* (Zeller, 1847) [**6**]

*vetustella* (Herrich-Schäffer, 1854)

*tripunctigerella* (Bruand d’Uzelle, 1859)

*submissella* (Frey, 1880), homonym

*rufobasella* (Rebel, 1916)

*yunusemrei* Koçak, 1986

*Stomopteryx spathulella* Nel, Varenne & Labonne, 2019 [**6**]

*Stomopteryx orthogonella* (Staudinger, 1871)

*Stomopteryx flavipalpella* Jäckh, 1959 [**7**]

*Stomopteryx hungaricella* Gozmány, 1957

*Stomopteryx lusitaniella* Corley & Karsholt, 2014

*Stomoptery jeppeseni* Karsholt & Šumpich, 2018

*Stomopteryx alpinella* Nel & Varenne, 2016

*Stomopteryx schizogynae* (Walsingham, 1908)

***Aproaerema* Durrant, 1897 [8**]

*Harpagus* Stephens, 1834, homonym

*Untomia* Busck, 1906

*Schuetzeia* Spuler, 1910

*Syncopacma* Meyrick, 1925

*Lixodessa* Gozmány, 1957

*Aproaerema patruella* (Mann, 1857)

*fulvistillella* (Rebel, 1891)

*Aproaerema coronillella* (Treitschke, 1833)

*fournieri* (Nel, 1998)

*Aproaerema incognitana* (Gozmány, 1957) **comb. nov. [8**]

*Aproaerema sangiella* (Stainton, 1863)

*Aproaerema cinctella* (Clerck, 1759) [**9**]

*vorticella* (Scopoli, 1763)

*ligulella* ([Denis & Schiffermüller], 1775)

*vittata* (Fourcroy & Geoffroy, 1785)

*vittatella* (Villers, 1789)

*albistrigella* (Stephens, 1834)

*ussuriella* (Caradja, 1920)

*finlandica* (Gozmány, 1957)

*Aproaerema cinctelloides* (Nel & Varenne, 2012) **comb. nov. [8**]

*Aproaerema larseniella* (Gozmány, 1957)

*ligulella* auct.

*Aproaerema wormiella* (Wolff, 1958) [**8**]

*parawormiella* (Nel & Varenne, 2016)

*Aproaerema azosterella* (Herrich-Schäffer, 1854) **comb. nov. [8**]

*Aproaerema ochrofasciella* (Toll, 1936)

*Aproaerema taeniolella* (Zeller, 1839)

*sircomella* (Stainton, 1854)

*Aproaerema montanata* (Gozmány, 1957) **comb. nov. [8**]

*Aproaerema albifrontella* (Heinemann, 1870)

*ignobilella* (Heinemann, 1870)

*Aproaerema cincticulella* (Bruand, 1851) **comb. nov.**

*Aproaerema vinella* Bankes, 1898

*fasciata* Bankes, 1898, unavailable

*biformella* Schütze, 1902

*Aproaerema buvati* (Nel, 1995) **comb. nov. [8**]

*Aproaerema linella* (Chrétien, 1904) **comb. nov. [8, 10**]

*schoenmanni* (Gozmány, 1957)

*Aproaerema albipalpella* (Herrich-Schäffer, 1854)

*leucopalpella* (Herrich-Schäffer, 1854), unavailable

*ruptella* (Constant, 1865)

*Aproaerema suecicella* (Wolff, 1958) [**11**]

*Aproaerema captivella* (Herrich-Schäffer, 1854) **comb. nov. [8**]

*sarothamnella* (Zeller, 1868)

*Aproaerema polychromella* (Rebel, 1902)

*argyrolobiella* Caradja, 1920, unavailable

*faceta* (Meyrick, 1914)

*Aproaerema karvoneni* (Hackman, 1950) [**12**]

*Aproaerema semicostella* (Staudinger, 1871) **comb. nov. [8**]

*albicapitella* (Bidzilya, 1996)

*Aproaerema steppicolella* (Junnilainen, 2010) **comb. nov. [8**]

*Aproaerema cottiennella* (Nel, 2012) **comb. nov. [8**]

*Aproaerema genistae* (Walsingham, 1908) **comb. rev. [8**]

*Aproaerema thaumalea* (Walsingham, 1905) **comb. rev. [8**]

*Aproaerema anthyllidella* (Hübner, 1813) [**13**]

*caliginosella* (Duponchel, 1843)

*elachistella* (Stainton, 1859), subspecies

*psoralella* (Millière, 1865)

*lachtensis* (Erschoff, 1877)

*sparsiciliella* (Barrett, 1891)

*infestella* (Rebel, 1896)

*natrixella* (Weber, 1945)

*brundini* (Benander, 1945)

*alfalfella* Amsel, 1958

*aureliana* Căpuşe, 1964

*Aproaerema lerauti* Vives, 2001

*Aproaerema mercedella* Walsingham, 1908

***Iwaruna* Gozmány, 1957 [14**]

*Iwaruna heringi* Gozmány, 1957

*Iwaruna biguttella* (Duponchel, 1843)

*Iwaruna klimeschi* Wolff, 1958

*Iwaruna robineaui* Nel, 2008

***Anacampsis* Curtis, 1827**

*Tachyptilia* Heinemann, 1870

*Agriastis* Meyrick, 1914

*Anacampsis populella* (Clerck, 1759) [**15**]

*tremella* ([Denis & Schiffermüller], 1775)

*boeberana* (Fabricius, 1787)

*populi* (Haworth, 1828), emendation

*laticinctella* Stephens, 1834

*tremulella* Duponchel, 1839

*atra* (Strand, 1901), unavailable

*lugens* (Caradja, 1920)

*sachalinensis* (Matsumura, 1931)

*fuscatella* (Bentinck, 1934)

*ambronella* (Meder, 1934)

*ceballosi* Agenjo, 1959

*Anacampsis blattariella* (Hübner, 1796) [**15**]

*thapsiella* (Hübner, 1796)

*blattariae* (Haworth, 1828), emendation

*atragriseella* Bruand d’Uzelle, 1851

*betulinella* Vári, 1941

*Anacampsis timidella* (Wocke, 1887)

*quercella* (Chrétien, 1907)

*disquei* (Meess, 1907)

*suberiella* Caradja, 1920

*Anacampsis scintillella* (Fischer v. Röslerstamm, 1841) [**16**]

*brunneella* Herrich-Schäffer, 1854

*contuberniella* (Staudinger, 1859)

*Anacampsis temerella* (Lienig & Zeller, 1846)

*pernigrella* (Douglas, 1850)

*Anacampsis trifoliella* (Constant, 1890)

*Anacampsis fuscella* (Eversmann, 1844)

*Anacampsis hirsutella* (Constant, 1885)

*Anacampsis obscurella* ([Denis & Schiffermüller], 1775) [**17**]

*subsequella* (Hübner, 1796)

*Anacampsis malella* Amsel, 1959

***Mesophleps* Hübner, 1825 [18**]

*Brachyacma* Meyrick, 1886

*Lathontogenus* Walsingham, 1897

*Paraspistes* Meyrick, 1905

*Chretienia* Spuler, 1910

*Lipatia* Busck, 1910

*Stiphrostola* Meyrick, 1923

*Crossobela* Meyrick, 1923

*Xerometra* Meyrick, 1925

*Gnosimacha* Meyrick, 1927

*Bucolarcha* Meyrick, 1929

*Uncustriodonta* Agenjo, 1952

*Mesophleps corsicella* (Herrich-Schäffer, 1856)

*lala* Agenjo, 1961

*Mesophleps silacella* (Hübner, 1796)

*pyropella* auct.

*luteella* (Hübner, 1896), unavailable

*silacea* (Haworth, 1828), emendation

*apicellus* Caradja, 1920

*calaritanus* Amsel, 1939

*Mesophleps oxycedrella* (Millière, 1871)

*Mesophleps trinotella* Herrich-Schäffer, 1856

*aurantiella* (Rebel, 1915)

*subtilipennis* (Turati, 1924)

*Mesophleps ochracella* (Turati, 1926)

*orientella* Nel & Nel, 2003

*gallicella* Varenne & Nel, 2011

**Chelariini Le Marchand, 1947**

Hypatimini Kloet & Hincks, 1945, unavailable

Anarsiini Amsel, 1977

***Nothris* Hübner, 1825 [19**]

*Nothris congressariella* (Bruand, 1858)

*declaratella* Staudinger, 1859

*Nothris lemniscellus* (Zeller, 1839)

*Nothris gregerseni* Karsholt & Šumpich, 2015 [**20**]

*Nothris verbascella* ([Denis & Schiffermüller], 1775)

*discretella* Rebel, 1889

*clarella* Amsel, 1935

*Nothris sulcella* Staudinger, 1879

*magna* Nel & Peslier, 2007

*Nothris radiata* (Staudinger, 1879) [**21**]

*Nothris skyvai* Karsholt & Šumpich, 2015

***Neofaculta* Gozmány, 1955**

*Haplovalva* Janse, 1958

*Neofaculta ericetella* (Geyer, 1832) [**22**]

*gallinella* (Treitschke, 1833)

*lanceolella* (Stephens, 1834)

*fuscella* (Duponchel, 1844)

*subatrella* (Duponchel, 1845)

*quinquemaculella* (Bruand d’Uzelle, 1859)

*orcella* (Zerny, 1927), subspecies

*atlanticella* (Amsel, 1938), subspecies

*tenalella* (Amsel, 1938)

*amseli* (Dufrane, 1955)

*pyrenemontana* (Dufrane, 1955)

*betulea* auct.

*Neofaculta infernella* (Herrich-Schäffer, 1854)

*infernalis*, unavailable

*Neofaculta taigana* Ponomarenko, 1998 [**23**]

***Hypatima* Hübner, 1825**

*Chelaria* Haworth, 1828

*Tituacia* Walker, 1864

*Stomylia* Snellen, 1878

*Allocota* Meyrick, 1904, homonym

*Cymatomorpha* Meyrick, 1904

*Deuteroptila* Meyrick, 1904

*Semodictis* Meyrick, 1909

*Allocotaniana* Strand, 1913

*Episacta* Turner, 1919

*Hypatima rhomboidella* (Linnaeus, 1758) [**24**]

*conscriptella* (Hübner, 1805)

*hubnerella* (Donovan, 1806), incorrect original spelling

*huebnerella* (Donovan, 1806), justified emendation

*conscripta* Haworth, 1828, emendation

***Anarsia* Zeller, 1839 [25**]

*Ananarsia* Amsel, 1959

*Anarsia lineatella* Zeller, 1839

*pullatella* (Hübner, 1796), nomen oblitum

*pruniella* Clemens, 1860

*heratella* Amsel, 1967, subspecies

*tauricella* Amsel, 1967, subspecies

*Anarsia innoxiella* Gregersen & Karsholt, 2017

*Anarsia spartiella* (Schrank, 1802)

*robertsonella* (Curtis, 1837)

*genistae* Stainton, 1854

*genistella* Doubleday, 1859, emendation

*ragonotella* Réal, 1994

*krausei* Réal, 1994

*lhommella* Réal, 1994

*acutiloba* Réal, 1994

*pseudospartiella* Réal, 1994

*ungemachi* Réal, 1994

*Anarsia bilbainella* (Rössler, 1877) [**26**]

*burmanni* Amsel, 1958

*bizensis* Réal, 1994

*infundiblulella* Réal, 1994

*ovilella* Réal, 1994

*Anarsia eleagnella* Kuznetsov, 1957

*Anarsia dejoannisi* Réal, 1994

*Anarsia leberonella* Réal, 1994

*Anarsia sibirica* Park & Ponomarenko, 1996

*Anarsia stepposella* Ponomarenko, 2002

*psammobia* Falkovitsh & Bidzilya, 2003

*Anarsia acaciae* Walsingham, 1896

*Anarsia balioneura* Meyrick, 1921

**Dichomeridinae Hampson, 1918**

Brachminae Omelko, 1999

Dichomerinae, misspelling

***Dichomeris* Hübner, 1818 [27**]

*Elasmion* Hübner, 1808, unavailable

*Oxybelia* Hübner, 1825

*Rhinosia* Treitschke, 1833

*Gaesa* Walker, 1864

*Uliaria* Dumont, 1921

*Cymotricha* Meyrick, 1923

*Acanthophila* Heinemann, 1870

*Mimomeris* Povolný, 1978

*Dichomeris acuminatus* (Staudinger, 1876)

*ianthes* (Meyrick, 1887)

*rusticus* (Walsingham, 1892)

*lotellus* (Constant, 1893)

*ammoxanthus* (Meyrick, 1904)

*ochrophanes* (Meyrick, 1907)

*sublotellus* (Caradja, 1920)

*Dichomeris cisti* (Staudinger, 1859)

*meridionella* (Walsingham, 1891)

*Dichomeris limbipunctellus* (Staudinger, 1859) [**28**]

*millierellus* Stainton, 1873

*Dichomeris neatodes* Meyrick, 1923 **sp. rev. [28**]

*Dichomeris helianthemi* (Walsingham, 1903)

*Dichomeris castellana* (Schmidt, 1941)

*Dichomeris juniperella* (Linnaeus, 1761) [**29**]

*juniperi* Haworth, 1828, emendation

*Dichomeris marginella* (Fabricius, 1781)

*fimbriella* (Thunberg, 1788)

*clarella* (Treitschke, 1833)

*Dichomeris ustalella* (Fabricius, 1794)

*capucinella* (Hübner, 1796)

*cornutus* (Fabricius, 1798)

*ustulatus* (Fabricius, 1798), emendation

*burgundiellus* (Bruand d’Uzelle, 1859)

*Dichomeris derasella* ([Denis & Schiffermüller], 1775)

*fasciella* (Hübner, 1796)

*unguiculatus* (Fabricius, 1798)

*coreanus* Matsumura, 1931

*paranthes* Meyrick, 1936

*Dichomeris limosellus* (Schläger, 1849)

*deflectivellus* (Reutti, 1853)

*Dichomeris nitiellus* (Costantini, 1923)

*Dichomeris rasilella* (Herrich-Schäffer, 1854) [**30**]

*lacrimella* (Caradja, 1920)

*insulella* (Dumont, 1921)

*occidentella* (Zerny, 1927), subspecies

*Dichomeris barbella* ([Denis & Schiffermüller], 1775)

*Dichomeris alacella* (Zeller, 1839)

*Dichomeris latipennella* (Rebel, 1937)

*scotosiella* (Hackman, 1945)

*piceana* (Šulcs, 1968)

*steueri* Povolný, 1978

***Anasphaltis* Meyrick, 1925**

*Anasphaltis renigerellus* (Zeller, 1839)

***Acompsia* Hübner, 1825 [31**]

*Brachycrossata* Heinemann, 1870

*Telephila* Meyrick, 1923

*Acompsia cinerella* (Clerck, 1759)

*murinella* (Scopoli, 1763)

*ardeliella* (Hübner, 1817)

*cinerea* (Haworth, 1828), emendation

*spodiella* (Treitschke, 1833)

*Acompsia pyrenaella* Huemer & Karsholt, 2002 [**32**]

*Acompsia antirrhinella* Millière, 1866 [**33**]

*Acompsia baldizzonei* Pinzari, Nel & Pinzari, 2016

*Acompsia maculosella* (Stainton, 1851) [**34**]

*Acompsia dimorpha* Petry, 1904

*Acompsia subpunctella* Svensson, 1966

*Acompsia delmastroella* Huemer, 1998

*Acompsia muellerrutzi* Wehrli, 1925

*Acompsia caucasella* Huemer & Karsholt, 2002

*Acompsia minorella* Rebel, 1899

*Acompsia tripunctella* ([Denis & Schiffermüller], 1775) [**35**]

*Acompsia ponomarenkoae* Huemer & Karsholt, 2002

*Acompsia schmidtiellus* (Heyden, 1848)

*durdhamellus* (Stainton, 1849)

*quadrinella* (Herrich-Schäffer, 1854)

***Brachmia* Hübner, 1825 [36**]

*Claododes* Heinemann, 1870, homonym

*Eudodacles* Snellen, 1889

*Aulacomima* Meyrick, 1904

*Apethistis* Meyrick, 1908

*Brachmia dimidiella* ([Denis & Schiffermüller], 1775) [**37**]

*costiguttella* (Lienig & Zeller, 1846)

*kneri* (Nowicki, 1864)

*Brachmia blandella* (Fabricius, 1798)

*gerronella* (Zeller, 1850)

*Brachmia procursella* Rebel, 1903

*Brachmia inornatella* (Douglas, 1850)

***Helcystogramma* Zeller, 1877**

*Ceratophora* Heinemann, 1870, homonym

*Dectobathra* Meyrick, 1904

*Teuchophanes* Meyrick, 1914

*Schemataspis* Meyrick, 1918

*Parelectra* Meyrick, 1925, homonym

*Psamathoscopa* Meyrick, 1937

*Anathyrsotis* Meyrick, 1939

*Parelectroides* Clarke, 1952

*Onebala* auct.

*Helcystogramma lineolella* (Zeller, 1839)

*Helcystogramma triannulella* (Herrich-Schäffer, 1854)

*sepiella* (Steudel, 1866)

*cinerea* (Caradja, 1931)

*macroscopa* (Meyrick, 1932)

*Helcystogramma lutatella* (Herrich-Schäffer, 1854)

*Helcystogramma rufescens* (Haworth, 1828)

*simplella* (Eversmann, 1844)

*diaphanella* (Lienig & Zeller, 1846)

*isabella* (Stainton, 1849)

*rufescentella* (Doubleday, 1859), emendation

*Helcystogramma albinervis* (Gerasimov, 1929)

*Helcystogramma arulensis* (Rebel, 1929)

*Helcystogramma klimeschi* Ponomarenko & Huemer, 2001

*Helcystogramma flavescens* Junnilainen, 2010

*Helcystogramma convolvuli* (Walsingham, 1908)

*chrypsilychna* (Meyrick, 1914)

*dryadopa* (Meyrick, 1918)

*effera* (Meyrick, 1918)

*emigrans* (Meyrick, 1921)

*Helcystogramma lamprostoma* (Zeller, 1847) [**38**]

*scutata* (Meyrick, 1894)

***Pseudosophronia* Corley, 2001 [39**]

*Pseudosophronia exustellus* (Zeller, 1847)

*catharurga* Meyrick, 1923

*parahumerella* Amsel, 1935

*buvati* Nel, 1998

*constanti* Nel, 1998, **syn. nov.**

*Pseudosophronia cosmella* (Constant, 1885)

**Apatetrinae Le Marchand, 1947**

Chrysoesthiinae Paclt, 1947, unavailable

**Pexicopiini Hodges, 1986**

***Harpagidia* Ragonot, 1895**

*Glaphyrerga* Meyrick, 1925

*Harpagidia magnetella* (Staudinger, 1871)

*pallidibasella* Ragonot, 1895

*melitophanes* (Meyrick, 1931)

***Pectinophora* Busck, 1917**

*Pectinophora gossypiella* (Saunders, 1844)

***Pexicopia* Common, 1958**

*Pexicopia malvella* (Hübner, 1805) [**40**]

*lutarea* (Haworth, 1828), unavailable

*umbrella* auct.

***Platyedra* Meyrick, 1895**

*Aratrognathosia* Gozmány, 1968, unavailable

*Platyedra subcinerea* (Haworth, 1828)

*vilella* (Zeller, 1847)

*parviocellatella* (Bruand d’Uzelle, 1851)

*bathrosticta* (Meyrick, 1937)

***Sitotroga* Heinemann, 1870**

*Nesolechia* Meyrick, 1921

*Syngenomictis* Meyrick, 1927

*Sitotroga psacasta* Meyrick, 1908

*celyphodes* (Meyrick, 1909)

*nea* Walsingham, 1920

*Sitotroga cerealella* (Olivier, 1789)

*hordei* (Kirby, 1815)

*arctella* (Walker, 1864)

*melanarthra* (Lower, 1900)

*palearis* (Meyrick, 1913)

*aenictopa* (Meyrick, 1927)

*ochrescens* (Meyrick, 1938)

*asemodes* (Meyrick, 1938)

**Apatetrini Le Marchand, 1947 [41**]

***Dactylotula* Cockerell, 1888**

*Dactylota* Snellen, 1876, homonym

*Didactylota* Walsingham, 1892

*Rotundivalva* Janse, 1951

*Dactylotula altithermella* (Walsingham, 1903)

*Dactylotula kinkerella* (Snellen, 1876) [**42**]

***Apatetris* Staudinger, 1879 [43**]

*Apatetris agenjoi* Gozmány, 1954

*Apatetris mediterranella* Nel & Varenne, 2012 [**44**]

***Catatinagma* Rebel, 1903**

*Catatinagma trivittellum* Rebel, 1903 [**45**]

*Catatinagma kraterella* Junnilainen & Nupponen, 2010 [**46**]

***Coloptilia* Fletcher, 1940**

*Colopteryx* Hofmann, 1898, homonym

*Coloptilia conchylidella* (Hofmann, 1898)

***Chrysoesthia* Hübner, 1825 [47**]

*Microsetia* Stephens, 1829

*Chrysia* Bruand d’Uzelle, 1851

*Nomia* Clemens, 1860, homonym

*Chrysopora* Clemens, 1860

*Nannodia* Heinemann, 1870

*Anaphaula* Walsingham, 1904

*Chrysoesthia drurella* (Fabricius, 1775) [**48**]

*myllerella* (Fabricius, 1794)

*zinckenlla* (Hübner, 1813)

*druryella* (Zeller, 1851), emendation

*hermannella* auct.

*Chrysoesthia eppelsheimi* (Staudinger, 1885)

*Chrysoesthia verrucosa* Tokár, 1999

*Chrysoesthia sexguttella* (Thunberg, 1794)

*auropunctella* (Thunberg, 1794)

*aurofasciella* (Stephens, 1834)

*naeviferella* (Duponchel, 1843)

*stipella* auct.

*Chrysoesthia halimionella* Bidzilya & Budashkin, 2015

*Chrysoesthia atriplicella* (Amsel, 1939) [**49**]

*Chrysoesthia gaditella* (Staudinger, 1859) [**49**]

*Chrysoesthia aletris* (Walsingham, 1919) [**49**]

*Chrysoesthia boseae* (Walsingham, 1908)

*Chrysoesthia falkovitshi* Lvovsky & Piskunov, 1989

*Chrysoesthia hispanica* Karsholt & Vives, 2014

***Metanarsia* Staudinger, 1871**

*Calyptrotis* Meyrick, 1891

*Epipararsia* Rebel, 1914

*Parametanarsia* Gerasimov, 1930

*Metanarsia modesta* Staudinger, 1871 [**50**]

*kurdistanella* Amsel, 1959, subspecies

*Metanarsia onzella* Christoph, 1887

*Metanarsia guberlica* Nupponen, 2010

*Metanarsia incertella* (Herrich-Schäffer, 1861)

*longivitella* (Rebel, 1914)

*halmyropis* (Meyrick, 1926)

*ramiferella* (Lucas, 1940)

***Oecocecis* Guenée, 1870**

*Oecocecis guyonella* Guenée, 1870 [**51**]

**Thiotrichinae Karsholt, Mutanen, Lee & Kaila, 2013 [52**]

Palumbininae Chapman, 1902, *nomen nudum*

***Thiotricha* Meyrick, 1886**

*Reuttia* Hofmann, 1898

*Mystax* Caradja, 1920, homonym

*Thiotricha majorella* Rebel, 1910

*Thiotricha subocellea* (Stephens, 1834)

*internella* (Lienig & Zeller, 1846)

*dissonella* (Herrich-Schäffer, 1854)

*subocellella* (Doubleday, 1859), emendation

*Thiotricha coleella* (Constant, 1885)

*Thiotricha wollastoni* (Walsingham, 1884)

***Palumbina* Rondani, 1876**

*Thyrsostoma* Meyrick, 1907

*Palumbina guerinii* (Stainton, 1858)

*terebintella* Rondani, 1876

*pistaciae* (Anagnostopoulos, 1935)

**Anomologinae Meyrick, 1926**

Aristoteliinae Le Marchand, 1947

Metzneriini Piskunov, 1975

Isophrictini Povolný, 1979

***Bryotropha* Heinemann, 1870 [53**]

*Mniophaga* Pierce & Daltry, 1938

*Adelphotropha* Gozmány, 1955

*Bryotropha sabulosella* (Rebel, 1905)

*Bryotropha domestica* (Haworth, 1828)

*domesticella* (Doubleday, 1859), emendation

*punctata* (Staudinger, 1876)

*salmonis* (Walsingham, 1908)

*algiricella* Chrétien, 1917

*Bryotropha vondermuhlli* Nel & Brusseaux, 2003

*Bryotropha rossica* Anikin & Piskunov, 1996

*tachengensis* Li & Zheng, 1997

*Bryotropha azovica* Bidzilia, 1997

*Bryotropha arabica* Amsel, 1952

*Bryotropha patockai* Elsner & Karsholt, 2003

*Bryotropha purpurella* (Zetterstedt, 1839)

*flavipalpella* (Nylander, 1848)

*Bryotropha tachyptilella* (Rebel, 1916)

*Bryotropha italica* Karsholt & Rutten, 2005

*Bryotropha politella* (Stainton, 1851)

*expolitella* (Doubleday, 1859)

*Bryotropha aliterrella* (Rebel, 1935)

*Bryotropha nupponeni* Karsholt & Rutten, 2005

*Bryotropha satschkovi* Anikin & Piskunov, 2018

*Bryotropha terrella* ([Denis & Schiffermüller], 1775) [**54**]

*inulella* (Hübner, 1805)

*pauperella* (Hübner, 1825)

*latella* (Herrich-Schäffer, 1854)

*lutescens* (Constant, 1865)

*suspectella* (Heinemann, 1870)

*alpicolella* Heinemann, 1870

*tenebrosella* (Teich, 1886)

*sardoterrella* Schawerda, 1936

*quignoni* Dufrane, 1938, unavailable

*joannisi* Dufrane, 1938, unavailable

*rufa* Dufrane, 1938, unavailable

*ochrea* Dufrane, 1938, unavailable

*Bryotropha sattleri* Nel, 2003

*Bryotropha desertella* (Douglas, 1850) [**55**]

*decrepidella* (Herrich-Schäffer, 1854)

*glabrella* Heinemann, 1870

*Bryotropha wolschrijni* Karsholt & Rutten, 2005

*Bryotropha heckfordi* Karsholt & Rutten, 2005

*Bryotropha figulella* (Staudinger, 1859)

*capnella* (Constant, 1865)

*cinnamomea* Turati, 1934

*Bryotropha plantariella* (Tengström, 1848)

*cinerosella* (Tengström, 1848)

*serrulatella* (Tengström, 1848)

*brevipalpella* Rebel, 1893

*Bryotropha galbanella* (Zeller, 1839)

*angustella* (Heinemann, 1870)

*ilmatariella* (Hoffmann, 1893)

*griseella* (Caradja, 1920)

*haareki* (Strand, 1920)

*fusconigratella* (Palm, 1947)

*Bryotropha boreella* (Douglas, 1851)

*Bryotropha sutteri* Karsholt & Rutten, 2005

*Bryotropha gallurella* Amsel, 1952

*Bryotropha hendrikseni* Karsholt & Rutten, 2005

*Bryotropha pallorella* Amsel, 1952

*mulinoides* Amsel, 1952

*zannonicola* Hartig, 1953

*Bryotropha hulli* Karsholt & Rutten, 2005 [**56**]

*Bryotropha plebejella* (Zeller, 1847)

*imperitella* (Staudinger, 1859)

*ancillula* (Walsingham, 1908)

*inexpectella* Nel, 1999

*Bryotropha dryadella* (Zeller, 1850)

*saralella* Amsel, 1952

*Bryotropha basaltinella* (Zeller, 1839)

*Bryotropha affinis* (Haworth, 1828) [**57**]

*tegulella* (Herrich-Schäffer, 1854)

*tectella* (Herrich-Schäffer, 1854)

*affinella* (Doubleday, 1859), emendation

*affinitella* (Bruand d’Uzelle, 1859), emendation

*Bryotropha umbrosella* (Zeller, 1839) [**58**]

*mundella* (Douglas, 1850)

*portlandicella* (Richardson, 1890)

*fulvipalpella* Joannis, 1908

*anacampsoidella* (Hering, 1924)

*oppositella* auct.

*Bryotropha similis* (Stainton, 1854)

*thuleella* (Zeller, 1857)

*similella* (Doubleday, 1859), emendation

*pullifimbriella* (Clemens, 1863)

*confinis* (Stainton, 1871)

*obscurecinerea* (Nolcken, 1871)

*stolidella* (Morris, 1872)

*fuliginosella* (Snellen, 1882)

*tahavusella* (Forbes, 1922)

*clandestina* (Meyrick, 1923)

*dufraneella* (Joannis, 1928)

*novisimilis* Li & Zheng, 1997

*Bryotropha senectella* (Zeller, 1839)

*ciliatella* (Herrich-Schäffer, 1854)

*obscurella* Heinemann, 1870

*minorella* Heinemann, 1870

*phoebusella* Millière, 1876

*larseni* Strand, 1927

***Epidola* Staudinger, 1859 [59**]

*Epidola stigma* Staudinger, 1859

*Epidola barcinonella* Millière, 1867

*Epidola semitica* Amsel, 1942 [**60**]

*Epidola nuraghella* Hartig, 1939

*Epidola melitensis* Amsel, 1955

***Aristotelia* Hübner, 1825 [61**]

*Ergatis* Heinemann, 1870, homonym

*Eucatoptus* Walsingham, 1897

*Aristotelia decurtella* (Hübner, 1813) [**62**]

*turbatella* (Treitschke, 1835)

*amoenella* (Joannis,1891)

*Aristotelia decoratella* (Staudinger, 1879)

*Aristotelia leonhardi* Krone, 1907

*Aristotelia ericinella* (Zeller, 1839) [**63**]

*silendrella* Caradja, 1920, unavailable

*Aristotelia subdecurtella* (Stainton, 1859) [**64**]

*Aristotelia subericinella* (Duponchel, 1843) [**65**]

*prohaskaella* (Rebel, 1907)

*Aristotelia billii* Varenne & Nel, 2013 [**66**]

*Aristotelia montarcella* Schmidt, 1941

*Aristotelia heliacella* (Herrich-Schäffer, 1854)

*rogenhoferi* (Staudinger, 1872)

*Aristotelia pancaliella* (Staudinger, 1871)

*Aristotelia baltica* Šulcs & Šulcs, 1983

*coeruleopictella* auct.

*Aristotelia brizella* (Treitschke, 1833)

*Aristotelia brizelloidea* Amsel, 1935

*Aristotelia confusella* Bidzilya & Budashkin, 2015

*Aristotelia staticella* Millière, 1876

*Aristotelia mirandella* Chrétien, 1908

*Aristotelia frankeniae* Walsingham, 1898

*Aristotelia calastomella* (Christoph, 1873)

*Aristotelia mirabilis* (Christoph, 1888)

***Caulastrocecis* Chrétien, 1931 [67**]

*Caulastrocecis pudicellus* (Mann, 1861)

*apicella* (Caradja, 1920)

*Caulastrocecis gypsella* (Constant, 1893)

*Caulastrocecis furfurella* (Staudinger, 1871) [**68**]

*Caulastrocecis cryptoxena* (Gozmány, 1952) **sp. rev. [68**]

*Caulastrocecis perexigella* Junnilainen, 2010

*Caulastrocecis interstratella* (Christoph, 1873)

*salinatrix* (Meyrick, 1926)

***Paranarsia* Ragonot, 1895 [69**]

*Paranarsia joannisiella* Ragonot, 1895

***Megacraspedus* Zeller, 1839 [70**]

*Chilopselaphus* Mann, 1867

*Chilopsephalus* Rebel, 1901, misspelling

*Toxoceras* Chrétien, 1915, homonym

*Toxidoceras* Chrétien, 1923

*Nevadia* Caradja, 1920, homonym

*Cauloecista* Dumont, 1928

*Reichardtiella* Filipjev, 1931

*Vadenia* Caradja, 1933

*Megacraspedus lanceolellus* (Zeller, 1850) [**71**]

*subdolellus* Staudinger, 1859

*hessleriellus* Rössler, 1868

*tutti* Walsingham, 1897

*grossisquammellus* Chrétien, 1925

*Megacraspedus bengtssoni* Huemer & Karsholt, 2018

*Megacraspedus junnilaineni* Huemer & Karsholt, 2018

*Megacraspedus uzunsyrtus* Bidzilya & Budashkin, 2015

*Megacraspedus similellus* Huemer & Karsholt, 2018

*Megacraspedus tokari* Huemer & Karsholt, 2018

*Megacraspedus dolosellus* (Zeller, 1839) [**72**]

*separatellus* (Fischer von Röslerstamm, 1843)

*incertellus* Rebel, 1930

*Megacraspedus neli* Huemer & Karsholt, 2018

*Megacraspedus faunierensis* Huemer & Karsholt, 2018

*Megacraspedus gredosensis* Huemer & Karsholt, 2018

*Megacraspedus cuencellus* Caradja, 1920

*Megacraspedus bidentatus* Huemer & Karsholt, 2018

*Megacraspedus fuscus* Huemer & Karsholt, 2018

*Megacraspedus trineae* Huemer & Karsholt, 2018

*Megacraspedus tristictus* Walsingham, 1910

*Megacraspedus alfacarellus* Wehrli, 1926

*Megacraspedus pusillus* Walsingham, 1903

*Megacraspedus skoui* Huemer & Karsholt, 2018

*Megacraspedus spinophallus* Huemer & Karsholt, 2018 [**73**]

*Megacraspedus occidentellus* Huemer & Karsholt, 2018

*Megacraspedus granadensis* Huemer & Karsholt, 2018

*Megacraspedus heckfordi* Huemer & Karsholt, 2018

*Megacraspedus tenuiuncus* Huemer & Karsholt, 2018

*Megacraspedus lativalvellus* Amsel, 1954

*Megacraspedus dejectella* (Staudinger, 1859)

*Megacraspedus devorator* Huemer & Karsholt, 2018

*Megacraspedus binotella* (Duponchel, 1843) [**74**]

*Megacraspedus brachypteris* Huemer & Karsholt, 2018 [**75**]

*Megacraspedus barcodiellus* Huemer & Karsholt, 2018

*Megacraspedus bilineatella* Huemer & Karsholt, 1996

*Megacraspedus andreneli* Varenne & Nel, 2014 [**76**]

*Megacraspedus sumpichi* Huemer & Karsholt, 2018

*Megacraspedus gallicus* Huemer & Karsholt, 2018

*Megacraspedus ribbeella* (Caradja, 1920)

*Megacraspedus numidellus* (Chrétien, 1915)

*mareotidellus* Turati, 1924,

*Megacraspedus albovenata* Junnilainen, 2010

*Megacraspedus longipalpella* Junnilainen, 2010

*Megacraspedus niphorrhoa* (Meyrick, 1926)

*Megacraspedus fallax* (Mann, 1867)

*Megacraspedus balneariellus* (Chrétien, 1907)

*Megacraspedus podolicus* (Toll, 1942)

*Megacraspedus knudlarseni* Huemer & Karsholt, 2018

*Megacraspedus imparellus* (Fischer v. Röslerstamm, 1843) [**77**]

*litovalvellus* Junnilainen, 2010

*Megacraspedus multispinella* Junnilainen & Nupponen, 2010

*Megacraspedus cerussatellus* Rebel, 1930

*Megacraspedus attritellus* Staudinger, 1871

*Megacraspedus lagopellus* (Herrich-Schäffer, 1860)

*Megacraspedus argyroneurellus* Staudinger, 1871

*Megacraspedus ibericus* Huemer & Karsholt, 2018

*Megacraspedus squalida* Meyrick, 1926

*escalerellus* Schmidt, 1941

*Megacraspedus pentheres* Walsingham, 1920

*Megacraspedus teriolensis* Huemer & Karsholt, 2018 [**78**]

*Megacraspedus korabicus* Huemer & Karsholt, 2018

*Megacraspedus quadristictus* Lhomme, 1946

*Megacraspedus eburnellus* Huemer & Karsholt, 2001

*Megacraspedus skulei* Huemer & Karsholt, 2018

*Megacraspedus peyerimhoffi* Le Cerf, 1925

*Megacraspedus peslieri* Huemer & Karsholt, 2018

***Dirhinosia* Rebel, 1905 [79**]

*Dirhinosia cervinella* (Eversmann, 1844)

*trifasciella* Rebel, 1905

*Dirhinosia arnoldiella* (Rebel, 1905)

*Dirhinosia interposita* Bidzilya & Budashkin, 2015

***Psamathocrita* Meyrick, 1925 [80**]

*Psamathocrita osseella* (Stainton, 1860)

*Psamathocrita argentella* Pierce & Metcalfe, 1942

*Psamathocrita dalmatinella* Huemer & Tokár, 2000

***Chimericorsa* Varenne, Huemer & Nel, 2017**

*Chimericorsa nioloensis* Varenne, Huemer & Nel, 2017

***Spiniphallellus* Bidzilya & Karsholt, 2008**

*Spiniphallellus desertus* Bidzilya & Karsholt, 2008

*Spiniphallellus chrysotosella* Junnilainen, 2016

***Deltophora* Janse, 1950**

*Deltophora maculata* (Staudinger, 1879)

*Deltophora stictella* (Rebel, 1927)

*Deltophora gielisia* Hull, 1995

***Ivanauskiella* Ivinskis & Piskunov, 1980 [81**]

*Spatuncusella* Nel & Varenne, 2013

*Ivanauskiella psamathias* (Meyrick, 1891)

*turkmenica* auct.

*Ivanauskiella occitanica* (Nel & Varenne, 2013) **sp. rev. [82**]

***Ptocheuusa* Heinemann, 1870 [83**]

*Syneunetis* Wallengren, 1881

*Ptocheuusa paupella* (Zeller, 1847) [**84**]

*inulella* (Curtis, 1850)

*melanolepidella* (Heydenreich, 1851)

*perniveella* (Bruand d’Uzelle, 1859)

*Ptocheuusa inopella* (Zeller, 1839) [**85**]

*amesella* Chrétien, 1908

*Ptocheuusa abnormella* (Herrich-Schäffer, 1854)

*Ptocheuusa minimella* (Rebel, 1936)

*Ptocheuusa asterisci* (Walsingham, 1903)

*Ptocheuusa scholastica* (Walsingham, 1903)

*Ptocheuusa guimarensis* (Walsingham, 1908)

*Ptocheuusa sublutella* Christoph, 1873

*Ptocheuusa cinerella* (Chrétien, 1908) **comb. nov. [86**]

***Gladiovalva* Sattler, 1960**

*Gladiovalva rumicivorella* (Millière, 1881)

*Gladiovalva aizpuruai* Vives, 1990

*Gladiovalva badidorsella* (Rebel, 1935)

***Ornativalva* Gozmány, 1955**

*Pelostola* Janse, 1960

*Ornativalva heluanensis* (Debski, 1913)

*frankeniivorella* (Chrétien, 1917)

*oasicolella* (Turati, 1924)

*siculella* (Mariani, 1937)

*Ornativalva ornatella* Sattler, 1967

*Ornativalva tamariciella* (Zeller, 1850)

*Ornativalva pseudotamariciella* Sattler, 1967

*Ornativalva antipyramis* (Meyrick, 1925)

*Ornativalva plutelliformis* (Staudinger, 1859)

*olbiaella* (Millière, 1861)

*siewersiellus* (Christoph, 1867)

*sinuatella* (Walsingham, 1904)

*Ornativalva sieversi* (Staudinger, 1871)

*Ornativalva mixolitha* (Meyrick, 1918)

*bipunctella* (Sattler, 1967), subspecies

***Atremaea* Staudinger, 1871**

*Calamotypa* Meyrick, 1926

*Atremaea lonchoptera* Staudinger, 1871

*exstans* (Meyrick, 1926)

***Amblypalpis* Ragonot, 1886 [87**]

*Amblypalpis olivierella* Ragonot, 1887

***Parapodia* Joannis, 1912 [88**]

*Cecidonostola* Amsel, 1958

*Parapodia sinaica* (Frauenfeld, 1860)

*tamaricicola* Joannis, 1912

*tamariciella* (Amsel, 1958)

***Isophrictis* Meyrick, 1917 [89**]

*Isophrictis robinella* (Chrétien, 1907)

*microlina* Meyrick, 1935

*Isophrictis meridionella* (Herrich-Schäffer, 1854)

*Isophrictis constantina* (Baker, 1888)

*Isophrictis cerdanica* Nel, 1995

*Isophrictis lineatellus* (Zeller, 1850)

*albilineella* (Bruand d´Uzelle, 1859)

*Isophrictis kefersteiniellus* (Zeller, 1850) [**90**]

*senicula* (Meyrick, 1913)

*Isophrictis striatella* ([Denis & Schiffermüller], 1775)

*tanacetella* (Schrank, 1802)

*substriatella* (Caradja, 1920), subspecies

*Isophrictis corsicella* Amsel, 1936

*Isophrictis invisella* (Constant, 1885)

*Isophrictis anthemidella* (Wocke, 1871) [**91**]

*Isophrictis impugnata* Gozmány, 1957

***Pyncostola* Meyrick, 1917**

*Pyncostola bohemiella* (Nickerl, 1864)

*tunesiella* (Chrétien, 1915)

*jablonkayi* (Gozmány, 1954)

***Metzneria* Zeller, 1839 [92**]

*Cleodora* Stephens, 1834, homonym

*Parasia* Duponchel, 1846

*Archimetzneria* Amsel, 1936

*Metzneria paucipunctella* (Zeller, 1839)

*zimmermanni* Hering, 1940

*confusalis* Lucas, 1956

*luqueti* Nel, 1995

*Metzneria tenuiella* (Mann, 1864)

*seminivora* (Walsingham, 1903)

*infelix* Walsingham, 1908

*insignificans* Walsingham, 1908

*Metzneria neuropterella* (Zeller, 1839)

*nevropterella* (Duponchel, 1843) [**93**]

*gigantella* Krulikowsky, 1909, unavailable

*Metzneria aestivella* (Zeller, 1839) [**94**]

*carlinella* (Stainton, 1851)

*selaginella* (Mann, 1855)

*torridella* (Mann, 1859)

*dichroa* Walsingham, 1908, subspecies.

*expositoi* Vives, 2001, **syn. nov.**

*Metzneria lappella* (Linnaeus, 1758)

*Metzneria castiliella* (Möschler, 1866)

*eatoni* Walsingham, 1899

*Metzneria littorella* (Douglas, 1850)

*quinquepunctella* (Herrich-Schäffer, 1854)

*Metzneria riadella* Englert, 1974

*Metzneria diffusella* Englert, 1974

*Metzneria fulva* Labonne, Huemer, Thibault & Nel, 2019 [**95**]

*Metzneria torosulella* (Rebel, 1893) [**95**]

*monochroa* Walsingham, 1908

*ignota* Turati, 1922

*Metzneria ehikeella* Gozmány, 1954 [**96**]

*Metzneria metzneriella* (Stainton, 1851) [**97**]

*falcatella* (Bruand d´Uzelle, 1859)

*Metzneria hilarella* Caradja, 1920

*Metzneria staehelinella* Englert, 1974

*Metzneria artificella* (Herrich-Schäffer, 1861) [**98**]

*litigiosella* (Millière, 1879)

*pannonicella* Rebel, 1915

*Metzneria agraphella* (Ragonot, 1895)

*incognita* Walsingham, 1904

*Metzneria aprilella* (Herrich-Schäffer, 1854) [**99**]

*igneella* (Tengström, 1859)

*sanguinolentella* Joannis, 1910

*Metzneria subflavella* Englert, 1974 [**100**]

*Metzneria filia* Piskunov, 1979

*Metzneria intestinella* (Mann, 1864)

*Metzneria santolinella* (Amsel, 1936)

*consimilella* Hackman, 1946

*Metzneria tristella* Rebel, 1901

*Metzneria campicolella* (Mann, 1857) [**101**]

*varennei* Nel, 1997

***Apodia* Heinemann, 1870**

*Apodia bifractella* (Duponchel, 1843)

*inulella* (Vallot, 1829), homonym

*Apodia martinii* Petry, 1911 **sp. rev. [102**]

***Pragmatodes* Walsingham, 1908 [103**]

*Pragmatodes fruticosella* Walsingham, 1908

*Pragmatodes melagonella* (Constant, 1895) **comb. nov. [103, 104**]

*Pragmatodes albagonella* (Varenne & Nel, 2010) **comb. nov. [103**]

*Pragmatodes cyrneogonella* (Nel & Varenne, 2012) **comb. nov. [103**]

*Pragmatodes parvulata* (Gozmány, 1953) **comb. nov. [103**]

*mediterranea* (Nel & Luquet, 1997)

***Argolamprotes* Benander, 1945**

*Argolamprotes micella* ([Denis & Schiffermüller], 1775)

*asterella* (Treitschke, 1833)

***Monochroa* Heinemann, 1870 [105**]

*Paltodora* Meyrick, 1894

*Catabrachmia* Rebel, 1909

*Monochroa rumicetella* (Hofmann, 1868) [**106**]

*acutangulella* (Heinemann, 1870)

*leptotechna* (Meyrick, 1937)

*Monochroa rebeli* (Hering, 1927)

*Monochroa sepicolella* (Herrich-Schäffer, 1854) [**107**]

*Monochroa rectifasciella* (Fuchs, 1902) [**107**]

*Monochroa tenebrella* (Hübner, 1817) [**108**]

*fuscocuprea* (Haworth, 1828)

*subcuprella* (Stephens, 1834)

*tenebrosella* (Zeller, 1839)

*parvella* (Heydenreich, 1851)

*fuscocuprella* Doubleday, 1859, emendation

*buffonella* (Millière, 1876)

*Monochroa scutatella* (Müller-Rutz, 1920)

*Monochroa dellabeffai* (Rebel, 1932)

*Monochroa servella* (Zeller, 1839) [**109**]

*farinosae* (Stainton,1867)

*Monochroa conspersella* (Herrich-Schäffer, 1854)

*questionella* (Herrich-Schäffer, 1854)

*morosa* (Mühlig, 1864)

*Monochroa tetragonella* (Stainton, 1885)

*gudmanni* (Larsen, 1927)

*Monochroa elongella* (Heinemann, 1870)

*micrometra* (Meyrick, 1935)

*Monochroa inflexella* Svensson, 1992

*Monochroa sperata* Huemer & Karsholt, 2010

*Monochroa lutulentella* (Zeller, 1839)

*brunickii* (Rebel, 1913)

*Monochroa aenigma* Anikin & Piskunov, 2018

*Monochroa saltenella* (Benander, 1928)

*Monochroa palustrellus* (Douglas, 1850)

*rozsikella* (Rebel, 1909)

*Monochroa divisella* (Douglas, 1850)

*csornensis* Rebel, 1909

*lepidolampra* (Gozmány, 1952)

*zarichella* Piskunov, 1975

*Monochroa lucidella* (Stephens, 1834) [**110**]

*scordiscella* (Rebel, 1904)

*unipunctella* (Amsel, 1935)

*immaculatella* Huemer, 1996, subspecies

*Monochroa simplicella* (Lienig & Zeller, 1846)

*impella* (Piskunov, 1975)

*Monochroa moyses* Uffen, 1991

*Monochroa arundinetella* (Boyd, 1857) [**111**]

*Monochroa suffusella* (Douglas, 1850) [**111**]

*oblitella* (Doubleday, 1859)

*peterseni* (Teich, 1901)

*Monochroa cytisella* (Curtis, 1837)

*fuscipennis* (Humphreys & Westwood, 1845)

*walkeriella* (Douglas, 1850)

*coenulentella* (Herrich-Schäffer, 1854)

*clinosema* (Meyrick, 1935)

*griseocapitella* (Bentinck, 1949), unavailable

*Monochroa ferrea* (Frey, 1870)

*latiuscula* (Heinemann, 1870)

*alfkeni* (Amsel, 1938)

*servella* auct.

*Monochroa nomadella* (Zeller, 1868) [**112**]

*Monochroa bronzella* Karsholt, Nel, Fournier, Varenne & Huemer, 2013

*Monochroa hornigi* (Staudinger, 1883)

*leptocrossa* (Meyrick, 1926)

*nordmanella* Bruun, 1958

*Monochroa niphognatha* (Gozmány, 1953)

***Oxypteryx* Rebel, 1911 [113**]

*Eulamprotes* Bradley, 1971

*Lamprotes* Heinemann, 1870, homonym

*Argyritis* Heinemann, 1870, homonym

*Siderea* Omelko, 1999

*Oxypteryx nigromaculella* (Millière, 1872) **comb. nov. [113, 114**]

*punctatella* (Staudinger, 1879)

*morphochroma* (Walsingham, 1900)

*jactatrix* (Meyrick, 1926)

*angustipennis* (Rebel, 1931)

*craterotypa* (Meyrick, 1939)

*donskoffi* (Nel & Luquet, 1997)

*Oxypteryx wilkella* (Linnaeus, 1758) **comb. nov. [113, 115**]

*merianella* (Linnaeus, 1758)

*germarella* (Geyer, 1832)

*pictella* (Zeller, 1839)

*tarquiniella* (Stainton, 1862)

*Oxypteryx ochricapilla* (Rebel, 1903) **comb. nov. [113**]

*Oxypteryx superbella* (Zeller, 1839) **comb. nov. [113**]

*Oxypteryx mirusella* (Huemer & Karsholt, 2013) **comb. nov. [113**]

*Oxypteryx baldizzonei* (Karsholt & Huemer, 2013) **comb. nov. [113, 116**]

*Oxypteryx occidentella* (Huemer & Karsholt, 2011) **comb. nov. [113**]

*Oxypteryx libertinella* (Zeller, 1872) **comb. nov. [113, 117**]

*Oxypteryx gemerensis* (Elsner, 2013) **comb. nov. [113**]

*Oxypteryx deserta* (Piskunov, 1990) **comb. nov. [113**]

*Oxypteryx unicolorella* (Duponchel, 1843) **comb. nov. [113**]

*lucentella* (Peyerimhoff, 1870)

*Oxypteryx atrella* ([Denis & Schiffermüller], 1775)

*quadripunctella* (Fabricius, 1781)

*umbriferella* (Herrich-Schäffer, 1854)

*aurimaculella* (Höfner, 1897)

*ornata* (Dufrane, 1942), unavailable

*Oxypteryx nigritella* (Zeller, 1847) **comb. nov. [113**]

*Oxypteryx immaculatella* (Douglas, 1850)

*phaeella* (Heckford & Langmaid, 1988)

*Oxypteryx plumbella* (Heinemann, 1870) **comb. nov. [113**]

*Oxypteryx isostacta* (Meyrick, 1926) **comb. nov. [113**]

*Oxypteryx helotella* (Staudinger, 1859) **comb. nov. [113**]

*damonella* (Millière, 1876)

*algeriella* (Baker, 1888)

*doliodes* (Meyrick, 1891)

*striatopunctella* (Rebel, 1891)

*levisella* (Chrétien, 1922)

*Oxypteryx parahelotella* (Nel, 1995) **comb. nov. [113**]

*Oxypteryx graecatella* (Šumpich & Skyva, 2012) **comb. nov. [113**]

**Gelechiinae Stainton, 1954**

**Gelechiini Stainton, 1954**

***Xystophora* Wocke, 1876**

*Doryphora* Heinemann, 1870, homonym

*Doryphorella* Cockerell, 1888

*Xystophora carchariella* (Zeller, 1839)

*Xystophora pulveratella* (Herrich-Schäffer, 1854)

*intaminatella* (Stainton, 1860)

*steudeliella* (Frey, 1880)

***Athrips* Billberg, 1820 [118**]

*Rhynchopacha* Staudinger, 1871

*Epithectis* Meyrick, 1895

*Leobatus* Walsingham, 1904

*Ziminiola* Gerasimov, 1930

*Cremona* Busck, 1934

*Athrips spiraeae* (Staudinger, 1871)

*Athrips pruinosella* (Lienig & Zeller, 1846)

*Athrips rancidella* (Herrich-Schäffer, 1854) [**119**]

*triatomaea* (Mühlig, 1864)

*vepretella* (Zeller, 1870)

*superfetella* (Peyerimhoff, 1877)

*cotoneastri* (Busck, 1934)

*haifella* Amsel, 1935

*cerasivorella* (Kuznetsov, 1960)

*Athrips thymifoliella* (Constant, 1893)

*Athrips amoenella* (Frey, 1882) [**120**]

*allgunnensis* Svensson, 1993, unavailable

*Athrips nigricostella* (Duponchel, 1842)

*Athrips tetrapunctella* (Thunberg, 1794)

*lathyri* (Stainton, 1865)

*lathyrella* (Doubleday, 1866), emendation

*Athrips mouffetella* (Linnaeus, 1758)

*pedisequella* ([Denis & Schiffermüller], 1775)

*punctifera* (Haworth, 1828)

*Athrips asarinella* (Chrétien, 1930)

*Athrips medjella* (Chrétien, 1900)

*Athrips patockai* (Povolný, 1979)

*Athrips polymaculella* Park, 1991

*Athrips stepposa* Bidzilya, 2005

*Athrips aquila* Junnilainen, 2010

*Athrips bidzilyai* Junnilainen, 2010

*Athrips fagoniae* (Walsingham, 1904)

***Neofriseria* Sattler, 1960**

*Neofriseria peliella* (Treitschke, 1835) [**121**]

*senecionella* (Bruand d´Uzelle, 1859)

*Neofriseria singula* (Staudinger, 1876)

*suppeliella* (Walsingham, 1896)

*amaurella* (Rebel, 1927), homonym

*ifranella* (Lucas, 1956)

*hispanicella* (Amsel, 1953)

*Neofriseria pseudoterrella* (Rebel, 1928)

*Neofriseria baungaardiella* Huemer & Karsholt, 1999

*Neofriseria hitadoella* Karsholt & Vives, 2014 [**122**]

*Neofriseria kuznetzovae* Bidzilya, 2002 [**123**]

*Neofriseria caucasicella* Sattler, 1960

*Neofriseria mongolinella* Piskunov, 1987

***Prolita* Leraut, 1993**

*Lita* Treitschke, 1833, homonym

*Prolita sexpunctella* (Fabricius, 1794)

*virgella* (Thunberg, 1794)

*longicornis* (Curtis, 1827)

*longicornella* (Doubleday, 1859), emendation

*histrionella* (Geyer, 1832)

*zebrella* (Treitschke, 1833)

*alpicolo* (Frey, 1867)

*alternatella* (Kearfott, 1908)

*melanica* (Strand, 1920), unavailable

*petulans* (Braun, 1925)

*Prolita solutella* (Zeller, 1839)

*fumosella* (Douglas, 1852)

*cornubiae* (Boyd, 1858)

*pribitzeri* (Rebel, 1889)

*nigrobipunctatella* (Lucas, 1932)

***Sophronia* Hübner, 1825 [124**]

*Sophronia semicostella* (Hübner, 1813) [**125**]

*marginella* (Thunberg, 1794), homonym

*Sophronia gelidella* Nordman, 1941

*Sophronia consanguinella* Herrich-Schäffer, 1854 [**126**]

*marginella* Toll, 1936

*Sophronia illustrella* (Hübner, 1796)

*Sophronia grandii* Hering, 1933 [**127**]

*ascalis* Gozmány, 1951, **syn. nov.**

*Sophronia chilonella* (Treitschke, 1833) [**128**]

*Sophronia finitimella* Rebel, 1905

*Sophronia acaudella* Rebel, 1903

*Sophronia curonella* Standfuss, 1884

*Sophronia humerella* ([Denis & Schiffermüller], 1775)

*Sophronia sicariellus* (Zeller, 1839) [**129**]

*Sophronia santolinae* Staudinger, 1863

***Mirificarma* Gozmány, 1955 [130**]

*Helina* Guenée, 1849, homonym

*Mirificarma rhodoptera* (Mann, 1866)

*Mirificarma minimella* Huemer & Karsholt, 2001

*Mirificarma denotata* Pitkin, 1984

*Mirificarma maculatella* (Hübner, 1796)

*Mirificarma aflavella* (Amsel, 1935)

*Mirificarma flavella* (Duponchel, 1844)

*segetella* (Zeller, 1847)

*Mirificarma eburnella* ([Denis & Schiffermüller], 1775)

*formosella* (Hübner, 1796), homonym

*flammella* (Hübner, 1825)

*rufeoformosella* (Bruand d´Uzelle, 1859)

*Mirificarma fasciata* Pitkin, 1984

*Mirificarma lentiginosella* (Zeller, 1839) [**131**]

*Mirificarma pederskoui* Huemer & Karsholt, 1999

*Mirificarma cytisella* (Treitschke, 1833) [**132**]

*roseella* (Hauder, 1918), unavailable

*leonella* Amsel, 1959, subspecies

*Mirificarma monticolella* (Rebel, 1931) [**133**]

*Mirificarma interrupta* (Curtis, 1827)

*interuptella* (Hübner, 1793), homonym

*Mirificarma burdonella* (Rebel, 1930) [**134**]

*Mirificarma cabezella* (Chrétien, 1925)

*Mirificarma ulicinella* (Staudinger, 1859) [**135**]

*Mirificarma mulinella* (Zeller, 1839)

*caminariella* (Fuchs, 1902)

*nigraesilvae* (Amsel, 1950)

***Aroga* Busck, 1914**

*Aroga velocella* (Zeller, 1839) [**136**]

*affiniella* (Zetterstedt, 1839)

*tesserella* (Zetterstedt, 1839)

*brunnea* (Schöyen, 1882)

*aterrimella* (Rebel, 1889)

*peperistis* (Meyrick, 1926)

*rupicolella* (Müller-Rutz, 1934)

*Aroga flavicomella* (Zeller, 1839) [**137**]

*aureodorsella* (Bruand d´Uzelle, 1859)

*Aroga eatoni* Corley & Goodey, 2014

*Aroga pascuicola* (Staudinger, 1871)

*eremella* (Chrétien, 1915)

*Aroga aristotelis* (Millière, 1876)

*astragali* (Staudinger, 1879)

*fulminella* (Millière, 1882)

*lacertella* (Walsingham, 1904)

*aplasticella* (Rebel, 1913), unavailable

*hyrcanella* (Toll, 1948)

*Aroga corsa* Varenne & Nel, 2019

*Aroga temporariella* Sattler, 1960

*Aroga balcanicola* Huemer & Karsholt, 1999

***Filatima* Busck, 1939**

*Filatima angustipennis* Sattler, 1961

*albicosta* auct.

*Filatima pallipalpella* (Snellen, 1884)

*Filatima spurcella* (Duponchel, 1843)

*fuscantella* (Heinemann, 1870)

*Filatima transsilvanella* Kovács & Kovács, 2002

*Filatima algarbiella* Corley, 2014

*Filatima tephritidella* (Duponchel, 1844)

*tephriditella* (Herrich-Schäffer, 1854)

*Filatima textorella* (Chrétien, 1908)

*Filatima djakovica* Anikin & Piskunov, 1996

*Filatima incomptella* (Herrich-Schäffer, 1854)

*turbidella* (Nolcken, 1871)

*Filatima ukrainica* Piskunov, 1971

*Filatima zagulajevi* Anikin & Piskunov, 1996

***Chionodes* Hübner, 1825 [138**]

*Chionodes lugubrella* (Fabricius, 1794)

*luctificella* (Hübner, 1813)

*lunatella* (Zetterstedt, 1839)

*Chionodes tragicella* (Heyden, 1865)

*libidinosa* (Staudinger, 1871)

*Chionodes soella* Huemer & Sattler, 1995

*Chionodes luctuella* (Hübner, 1793) [**139**]

*sauteriella* (Zeller, 1868)

*Chionodes aprilella* Huemer & Sattler, 1995

*Chionodes violacea* (Tengström, 1848)

*Chionodes mongolica* Piskunov, 1979

*ukrainica* Piskunov, 1979

*Chionodes holosericella* (Herrich-Schäffer, 1854)

*cognatella* (Heinemann, 1870)

*norvegiae* (Strand, 1903)

*dovrella* (Grønlien, 1925)

*meesi* (Barca, 1932)

*danieli* (Osthelder, 1951)

*Chionodes praeclarella* (Herrich-Schäffer, 1854)

*pergrandella* (Rebel, 1917)

*colorella* (Caradja, 1920), unavailable

*decolorella* auct.

*Chionodes caucasicella* Huemer & Sattler, 1995

*Chionodes nubilella* (Zetterstedt, 1839)

*tarandella* (Wocke, 1864)

*Chionodes continuella* (Zeller, 1839)

*brumella* (Clemens, 1864)

*trimaculella* (Packard, 1867)

*albomaculella* (Chambers, 1875)

*Chionodes perpetuella* (Herrich-Schäffer, 1854)

*Chionodes apolectella* (Walsingham, 1900)

*Chionodes distinctella* (Zeller, 1839)

*striolatella* (Heinemann, 1870)

*tristella* (Teich, 1889)

*indistinctella* (Rebel, 1901)

*latiorella* (Amsel, 1939)

*unicolor* (Toll, 1948)

*deserticola* Piskunov, 1979

*Chionodes hayreddini* Koçak, 1986

*ochripalpella* (Frey, 1880), homonym

*Chionodes hinnella* (Rebel, 1935)

*Chionodes bastuliella* (Rebel, 1931)

*Chionodes electella* (Zeller, 1839)

*Chionodes viduella* (Fabricius, 1794)

*leucomella* (Quenzel, 1802)

*luctiferella* (Herrich-Schäffer, 1856)

*labradoriella* (Clemens, 1863)

*Chionodes nebulosella* (Heinemann, 1870)

*Chionodes fumatella* (Douglas, 1850) [**140**]

*celerella* (Stainton, 1851)

*oppletella* (Herrich-Schäffer, 1854)

*reuttiella* (Heinemann, 1870)

*nigricans* (Heinemann, 1870)

*syrticola* (Staudinger, 1871)

*brunnea* (Teich, 1901), homonym

*carpella* Piskunov, 1971

*Chionodes ignorantella* (Herrich-Schäffer, 1854)

*ochrisignella* (Nolcken, 1871)

***Gelechia* Hübner, 1825 [141**]

*Guenea* Bruand d´Uzelle, 1851

*Cirrha* Chambers, 1872

*Oeseis* Chambers, 1875

*Mesogelechia* Omelko, 1986

*Gelechia rhombella* ([Denis & Schiffermüller], 1775)

*rhombea* (Haworth, 1828), emendation

*axilella* (Thunberg, 1794)

*Gelechia scotinella* Herrich-Schäffer, 1854

*conspurcatella* Heinemann, 1870

*confusella* Heinemann, 1870

*kiesenwetteri* Heuäcker, 1873

*lakatensis* Rebel, 1904

*baueri* (Rebel, 1917)

*Gelechia senticetella* (Staudinger, 1859) [**142**]

*limitanella* Rebel, 1904

*nigrostriella* (Zerny, 1936)

*Gelechia obscuripennis* (Frey, 1880) [**143**]

*melanotica* (Burmann, 1950), unavailable

*albicans* (Burmann, 1950), unavailable

*Gelechia sabinellus* (Zeller, 1839)

*hoffmanniella* (Strand, 1902)

*corsella* (Rebel, 1930)

*kalevalella* (Kanerva, 1936)

*Gelechia atlanticella* (Amsel, 1955)

*Gelechia nervosella* (Zerny, 1927)

*thuriferella* (Cleu, 1936)

*Gelechia sororculella* (Hübner, 1817)

*Gelechia jakovlevi* Krulikovsky, 1905

*nigrovittata* Schantz, 1971

*Gelechia muscosella* Zeller, 1839

*griseella* Caradja, 1920

*Gelechia cuneatella* Douglas, 1852

*Gelechia aspoecki* Huemer, 1992

*Gelechia asinella* (Hübner, 1796)

*aurorella* Frey, 1882

*Gelechia hippophaella* (Schrank, 1802)

*basalis* Stainton, 1854

*acupediella* Frey, 1870

*Gelechia basipunctella* Herrich-Schäffer, 1854

*basiguttella* Heinemann, 1870

*albicans* Heinemann, 1870

*Gelechia nigra* (Haworth, 1828)

*cautella* Zeller, 1839

*Gelechia turpella* ([Denis & Schiffermüller], 1775)

*populella* (Hübner, 1796)

*nebulea* (Haworth, 1828), unavailable

*pinguinella* (Treitschke, 1832)

*kochiella* (Herrich-Schäffer, 1854)

*Gelechia rhombelliformis* Staudinger, 1871

*Gelechia sirotina* Omelko, 1986

*Gelechia sestertiella* Herrich-Schäffer, 1854

*Gelechia mediterranea* Huemer, 1991

*Gelechia dujardini* Huemer, 1991

***Psoricoptera* Stainton, 1854**

*Psoricoptera speciosella* Teich, 1893

*Psoricoptera gibbosella* (Zeller, 1839)

*triorthias* (Meyrick, 1935)

*lepigreella* (Lucas, 1938)

***Agnippe* Chambers, 1872 [144**]

*Evippe* Chambers, 1873

*Phaetusa* Chambers, 1875, homonym

*Tholerostola* Meyrick, 1917

*Agnippe echinuloides* Bidzilya & Li, 2010

*Agnippe lunaki* (Rebel, 1941)

*penicillata* (Amsel, 1961)

*Agnippe pseudolella* (Christoph, 1888)

*cephalella* (Caradja, 1920)

***Holcophora* Staudinger, 1871 [145**]

*Aponoaea* Walsingham, 1905

*Holcophora statices* Staudinger, 1871

*Holcophora inderskella* (Caradja, 1920) [**146**]

*Holcophora obtusipalpis* (Walsingham, 1905)

*cinerellus* (Turati, 1930)

**Gnorimoschemini Povolný, 1964**

***Gnorimoschema* Busck, 1900**

*Lerupsia* Riedl, 1965

*Neoschema* Povolný, 1967

*Gnorimoschema soffneri* (Riedl, 1965)

*antiquum* Povolný, 1966

*Gnorimoschema herbichii* (Nowicki, 1864) [**147**]

*pusillella* (Rebel, 1893)

*tengstroemiella* (Joannis, 1910)

*pazsiczkyi* (Rebel, 1913)

*parentesella* (Toll, 1936)

*tengstroemi* (Hackman, 1946)

*mongoliae* Povolný, 1973, subspecies

*kamchaticum* Povolný, 1977, subspecies

*Gnorimoschema bodillum* Karsholt & Nielsen, 1974

*Gnorimoschema nupponeni* Huemer & Karsholt, 2010

*Gnorimoschema robustella* (Staudinger, 1871)

*syrphetopa* (Meryick, 1926)

*Gnorimoschema steueri* Povolný, 1975

*Gnorimoschema epithymella* (Staudinger, 1859)

*brunneomaculella* (Hackman, 1946), subspecies

*boerneri* (Amsel, 1952), subspecies

*kirgisicum* Povolný, 1994, subspecies

*Gnorimoschema nordlandicolella* (Strand, 1902)

*cyceonodes* (Meyrick, 1924)

*eucausta* (Meyrick, 1929)

*fennicella* (Hackman, 1946)

*Gnorimoschema nilsi* Huemer, 1996

*Gnorimoschema valesiella* (Staudinger, 1877)

*diabolicella* (Hering, 1924)

*charcoti* (Meyrick, 1934)

*hackmani* (Schantz, 1952)

*Gnorimoschema streliciella* (Herrich-Schäffer, 1854)

*Gnorimoschema hoefneri* (Rebel, 1909)

***Scrobipalpopsis* Povolný, 1967**

*Scrobipalpopsis petasitis* (Pfaffenzeller, 1867)

*petasitella* (Staudinger, 1867)

*petasitae* (Heinemann, 1870), emendation

***Tecia* Povolný, 1973**

*Tecia solanivora* (Povolný, 1973)

***Scrobipalpa* Janse, 1951 [148**]

*Ilseopsis* Povolný, 1965

*Euscrobipalpa* Povolný, 1967

*Ergasiola* Povolný, 1967

*Scrobipalpa aptatella (Walker, 1864) [**149**]

*heliopa* (Lower, 1900)

*Scrobipalpa kasyi* Povolný, 1968

*Scrobipalpa notata* (Povolný, 2001)

*Scrobipalpa acuminatella* (Sircom, 1850)

*pulliginella* (Sircom, 1850)

*cirsiella* (Stainton, 1851)

*porcella* (Heinemann, 1870)

*ingloriella* (Heinemann, 1870)

*gracilella* (Stainton, 1871)

*Scrobipalpa skulei* Huemer & Karsholt, 2010

*Scrobipalpa hungariae* (Staudinger, 1871)

*Scrobipalpa adaptata* (Povolný, 2001)

*Scrobipalpa brahmiella* (Heyden, 1862)

*Scrobipalpa vasconiella* (Rössler, 1877)

*drahomirae* Povolný, 1966

*Scrobipalpa dorsolutea* Huemer & Karsholt, 2010

*Scrobipalpa amseli* Povolný, 1966 [**150**]

*Scrobipalpa hyssopi* Nel, 2003 [**150**]

*Scrobipalpa montanella* (Chrétien, 1910)

*Scrobipalpa corleyi* Huemer & Karsholt, 2010

*Scrobipalpa chrysanthemella* (Hofmann, 1867)

*opificella* (Mann, 1878)

*Scrobipalpa proclivella* (Fuchs, 1886)

*rancidella* auct.

*Scrobipalpa frugifera* Povolný, 1969

*hypothetica* Povolný, 1973

*Scrobipalpa oleksiyella* Huemer & Karsholt, 2010

*Scrobipalpa smithi* Povolný & Bradley, 1964

*Scrobipalpa occulta* (Povolný, 2002)

*sibirica* Bidzilya, 2009

*Scrobipalpa grisea* Povolný, 1969

*uralensis* Povolný, 1973, unavailable

*Scrobipalpa usingeri* Povolný, 1969

*Scrobipalpa clintoni* Povolný, 1968

*linella* Piskunov, 1975

*deleta* Povolný, 1981

*Scrobipalpa reiprichi* Povolný, 1984 [**151**]

*Scrobipalpa obsoletella* (Fischer v. Röslerstamm, 1841)

*miscitatella* (Clarke, 1932)

*bipunctella* (Hartig, 1941)

*calaritanella* (Amsel, 1952)

*hospes* Povolný, 1964

*Scrobipalpa feralella* (Zeller, 1872)

*rebeliella* (Hauder, 1917)

*Scrobipalpa halonella* (Herrich-Schäffer, 1854)

*Scrobipalpa perinii* (Klimesch, 1951)

*Scrobipalpa phagnalella* (Constant, 1895)

*staehelinella* (Caradja, 1920), unavailable

*Scrobipalpa tokari* Huemer & Karsholt, 2010

*Scrobipalpa karadaghi* (Povolný, 2001)

*Scrobipalpa heimi* Huemer & Karsholt, 2010

*Scrobipalpa acuta* (Povolný, 2001)

*Scrobipalpa soffneri* Povolný, 1964

*Scrobipalpa jariorum* Huemer & Karsholt, 2010

*Scrobipalpa murinella* (Duponchel, 1843)

*culminicolella* (Staudinger, 1871)

*excelsa* (Frey, 1880)

*Scrobipalpa wiltshirei* Povolný, 1966

*obrteliana* Povolný, 1971, subspecies

*Scrobipalpa caucasica* (Povolný, 2001) [**152**]

*bezengensis* (Povolný, 2001)

*Scrobipalpa pauperella* (Heinemann, 1870) [**153**]

*klimeschi* Povolný, 1967

*Scrobipalpa spumata* (Povolný, 2001)

*Scrobipalpa arenbergeri* Povolný, 1973

*Scrobipalpa mercantourica* Varenne & Nel, 2018 [**154**]

*Scrobipalpa nana* Povolný, 1973

*caroxyli* (Falkovitsh & Bidzilya, 2006), subspecies

*Scrobipalpa heretica* Povolný, 1973

*submagnificella* Povolný, 1977

*Scrobipalpa bigoti* Povolný, 1973

*tunesica* Povolný, 1979, subspecies

*Scrobipalpa dorsoflava* (Povolný, 1996)

*Scrobipalpa magnificella* Povolný, 1967

*Scrobipalpa abstrusa* Huemer & Karsholt, 2010

*Scrobipalpa superstes* Povolný, 1977

*Scrobipalpa remota* Povolný, 1972

*Scrobipalpa plesiopicta* Povolný, 1969

*Scrobipalpa bradleyi* Povolný, 1971

*glaserorum* Povolný, 1977

*meyricki* auct.

*Scrobipalpa selectella* (Caradja, 1920)

*fraterna* Povolný, 1969

*Scrobipalpa alterna* (Falkovitsh & Bidzilya, 2006) [**155**]

*Scrobipalpa lutea* Povolný, 1977 [**155**]

*Scrobipalpa griseoflava* Bidzilya & Budashkin, 2011

*Scrobipalpa niveifacies* Povolný, 1977

*milleri* Povolný, 1977

*Scrobipalpa indignella* (Staudinger, 1879)

*pseudobsoletellum* (Povolný & Gregor, 1955)

*hyoscyamivora* (Gerasimov, 1940)

*grossa* Povolný, 1966

*Scrobipalpa punctata* (Povolný, 1996)

*Scrobipalpa lagodes* (Meyrick, 1926)

*Scrobipalpa deluccae Povolný, 1966

*Scrobipalpa atriplicella* (Fischer von Röslerstamm, 1841)

*atrella* (Thunberg, 1788), homonym

*detersella* (Clemens, 1860), homonym

*infumatella* (Fuchs, 1901)

*brackenridgiella* (Busck, 1903)

*chenopodiella* (Busck, 1916)

*arogantella* Povolný, 1967

*altajica* Povolný, 1969

*Scrobipalpa suaedella* (Richardson, 1893)

*flavidorsella* (Amsel, 1952)

*hartigi* Povolný, 1977

*Scrobipalpa solitaria* Povolný, 1969

*Scrobipalpa dagmaris Povolný, 1987

*rezniki* Piskunov, 1990

*turkmenica* Piskunov, 1990

*Scrobipalpa suasella* (Constant, 1895)

*Scrobipalpa hendrikseni* Huemer & Karsholt, 2010

*Scrobipalpa halimifolia* Bidzilya & Budashkin, 2011

*Scrobipalpa traganella* (Chrétien, 1915)

*Scrobipalpa bazae* Povolný, 1977

*Scrobipalpa artemisiella* (Treitschke, 1833) [**156**]

*ancillella* (Bruand d’Uzelle, 1851)

*paniculatella* (Novickij, 1924)

*mongolensis* Povolný, 1969

*oreocyrniella* (Petry, 1904), subspecies

*syriaca* Povolný, 1967, subspecies

*Scrobipalpa stangei* (Hering, 1889) [**156**]

*saltenella* (Meess, 1910)

*Scrobipalpa suaedivorella* (Chrétien, 1915)

*detersipunctella* (Toll, 1947)

*Scrobipalpa bryophiloides* Povolný, 1966 [**157**]

*Scrobipalpa algeriensis* Povolný & Bradley, 1964

*Scrobipalpa deutschi* Huemer & Karsholt, 2010

*Scrobipalpa disjectella* (Staudinger, 1859)

*Scrobipalpa fontanensis* Varenne & Nel, 2017

*Scrobipalpa mixta* Huemer & Karsholt, 2010

*Scrobipalpa achtubica* Anikin & Piskunov, 2018

*Scrobipalpa rebeli* (Preissecker, 1914)

*fuscella* (Klimesch, 1938)

*japonica* Povolný, 1977

*Scrobipalpa gallicella* (Constant, 1885)

*Scrobipalpa ustulatella* (Staudinger, 1871)

*Scrobipalpa postulatella* Huemer & Karsholt, 2010

*Scrobipalpa filia* Povolný, 1969

*Scrobipalpa nitentella* (Fuchs, 1902)

*seminella* (Pierce & Metcalfe, 1935)

*Scrobipalpa costella* (Humphreys & Westwood, 1845)

*costimaculella* (Bruand d´Uzelle, 1859)

*Scrobipalpa hyoscyamella* (Stainton, 1869)

*Scrobipalpa portosanctana* (Stainton, 1859)

*eremaula* (Meyrick, 1891)

*lyciella* (Walsingham, 1900)

*desertella* (Rebel, 1901)

*bertramella* (Lucas, 1940)

*leroyella* (Lucas 1950)

*reisseri* (Povolný & Gregor, 1955)

*philolycii* (Hering, 1957)

*gallincolella* auct.

*Scrobipalpa vicaria* (Meyrick, 1921)

*tineiformis* Povolný, 1967

*Scrobipalpa ocellatella* (Boyd, 1858) [**158**]

*ocellatella* (Stainton, 1859), homonym

*submissella* (Stainton, 1859)

*horticolella* (Rössler, 1866)

*clarella* (Caradja, 1920)

*obscurior* (Rebel, 1927)

*orientale* (Gregor & Povolný, 1954)

*portosanctana* auct.

*Scrobipalpa pulchra* Povolný, 1967

*Scrobipalpa gecko* (Walsingham, 1911)

*Scrobipalpa hannemanni* Povolný, 1966

*furva* Povolný, 1969, subspecies

*gamanthi* (Falkovitsh & Bidzilya, 2006), subspecies

*Scrobipalpa erichi* Povolný, 1964

*Scrobipalpa divisella* (Rebel, 1936)

*Scrobipalpa voltinella* (Chrétien, 1898)

*Scrobipalpa corsicamontes* Varenne & Nel, 2013

*Scrobipalpa suaedicola* (Mabille, 1906)

*suaedicola* (Amsel, 1939), homonym

*mabillei* Povolný, 1971

*Scrobipalpa monochromella* (Constant, 1895)

*Scrobipalpa samadensis* (Pfaffenzeller, 1870)

*plantaginella* (Stainton, 1883)

*brunhildae* (Schawerda, 1921)

*zimmermanni* (Zimmermann, 1923), unavailable

*mariae* (Zimmermann, 1926)

*testacella* (Rebel, 1935)

*echo* (Meyrick, 1937)

*Scrobipalpa salinella* (Zeller, 1847) [**159**]

*omachella* auct.

*zernyella* (Rebel, 1918)

*corsicanum* (Gregor & Povolný, 1954)

*ignotum* (Gregor & Povolný, 1954)

*trebujenae* Povolný, 1977

*Scrobipalpa spergulariella* (Chrétien, 1910) [**159**]

*Scrobipalpa salicorniae* (Hering, 1889) [**159**]

*caliacrae* (Caradja, 1932)

*Scrobipalpa halimioniella* Huemer & Karsholt, 2010

*Scrobipalpa thymelaeae* (Amsel, 1939)

*Scrobipalpa halymella* (Millière, 1864) [**160**]

*Scrobipalpa camphorosmella* Nel, 1999

*Scrobipalpa stabilis* Povolný, 1977 [**160**]

*Scrobipalpa instabilella* (Douglas, 1846)

*lagunella* (Chrétien, 1910)

*strobilacella* (Caradja, 1920), unavailable

*salsolella* (Amsel, 1935)

*halymiphaga* (Amsel, 1952)

*Scrobipalpa peterseni* (Povolný, 1965)

*Scrobipalpa ergasima* (Meyrick, 1916)

*hyoscyamella* (Rebel, 1912), homonym

*mignatella* (Caradja, 1920), unavailable

*intestina* (Meyrick, 1921)

*mirabile* (Gregor & Povolný, 1955)

*pervada* (Clarke, 1962)

***Turcopalpa* Povolný, 1973**

*Turcopalpa glaseri* Povolný, 1973

***Scrobipalpula* Povolný, 1964 [161**]

*Scrobipalpula psilella* (Herrich-Schäffer, 1854)

*nocturnella* (Staudinger, 1859)

*pallidella* (Heinemann, 1870)

*killiasii* (Frey, 1880)

*astericolellum* (Hering, 1957), unavailable

*asiatica* Povolný, 1968, subspecies

*Scrobipalpula ramosella* (Müller-Rutz, 1934)

*Scrobipalpula seniorum* Povolný, 2000

*ptarmicae* (Hering, 1957), unavailable

*compositella* (Povolný, 1964), unavailable

*Scrobipalpula diffluella* (Frey, 1870)

*cacuminum* (Frey, 1870)

*diffluella* (Heinemann, 1870)

*bellidiastri* (Klimesch, 1951)

*uniflorellum* (Hering, 1957), unavailable

*Scrobipalpula tussilaginis* (Stainton, 1867)

*tussilaginella* (Heinemann, 1870)

*retusella* (Rebel, 1891)

***Phthorimaea* Meyrick, 1902**

*Phthorimaea operculella* (Zeller, 1873)

*terrella* (Walker, 1864)

*solanella* (Boisduval, 1874)

*tabacella* (Ragonot, 1879)

*sedata* (Butler, 1880)

*argentinae* Povolný, 1989

*piscipellis* auct.

*epicentra* auct.

***Tuta* Kieffer & Jørgensen, 1910**

*Tuta absoluta* (Meyrick, 1917)

***Keiferia* Busck, 1939 [162**]

*Keiferia lycopersicella* (Walsingham, 1897)

***Ephysteris* Meyrick, 1908**

*Microcraspedus* Janse, 1958

*Opacopsis* Povolný, 1964

*Echinoglossa* Clarke, 1965

*Ephysteris promptella* (Staudinger, 1859) [**163**]

*despectella* (Walker, 1863)

*petiginella* (Mann, 1867)

*parvula* (Staudinger, 1879)

*cacomicra* (Walsingham, 1908)

*chersaea* Meyrick, 1908

*oschophora* (Meyrick, 1910)

*crystallista* (Meyrick, 1911)

*dispensata* (Meyrick, 1921)

*fanatica* (Meyrick, 1921)

*xanthorhabda* (Gozmány, 1951)

*australiae* Povolný, 1977

*Ephysteris tenuisaccus* Nupponen, 2010

*Ephysteris deserticolella* (Staudinger, 1871)

*albocapitella* (Rebel, 1928)

*buvati* (Povolný, 1992)

*Ephysteris insulella* (Heinemann, 1870)

*insularis* (Staudinger, 1871)

*praticolella* (Christoph, 1872), subspecies

*gallica* (Povolný, 1992)

*Ephysteris brachyptera* Karsholt & Sattler, 1998

*Ephysteris diminutella* (Zeller, 1847) [**164**]

*lunaki* (Hartig, 1941)

*treskensis* Povolný, 1964

*hispanica* Povolný, 1981

*foulonsensis* Povolný, 1981

*Ephysteris inustella* (Zeller, 1839) [**165**]

*delminiella* (Rebel, 1904)

*gredosensis* (Rebel, 1935), subspecies

*Ephysteris olympica* Povolný, 1968

*monticola* Povolný, 1981

*Ephysteris iberica* Povolný, 1977

***Ochrodia* Povolný, 1966 [166**]

*Ochrodia subdiminutella* (Stainton, 1867)

*jamaicensis* (Walsingham, 1897)

*bucolica* (Meyrick, 1904)

*zygophyllella* (Rebel, 1912)

*ericnista* (Meyrick, 1914)

*ferritincta* (Turner, 1919), subspecies

*ochrodeta* (Meyrick, 1923)

*extorris* (Meyrick, 1923)

*crocoleuca* (Meyrick, 1923)

*unitella* (Turati, 1930)

*tribulivora* (Dumont, 1931)

*pulverea* (Janse, 1950)

*turgida* (Janse, 1951)

*pentamacula* (Janse, 1958)

*infallax* (Gozmány, 1960)

*tractatum* (Gozmány, 1960)

***Vladimirea* Povolný, 1967**

*Distinxia* Povolný, 1967

*Vladimirea glebicolorella* (Erschoff, 1874)

*submaculata* Povolný, 1967

***Microlechia* Turati, 1924**

*Hedma* Dumont, 1932

*Megalocypha* Janse, 1960

*Microlechia rhamnifoliae* (Amsel & Hering, 1931)

*rhamnifoliae* (Amsel, 1935)

*Microlechia chretieni* Turati, 1924

*microcasis* (Meyrick, 1929)

*micradelpha* (Walsingham, 1900), homonym

*hyoscyamella* (Amsel & Hering, 1931), homonym

*abzacella* (Dumont, 1932)

*hyoscyami* (Amsel, 1935)

*polioptera* (Janse, 1960)

*aellographa* (Janse, 1960)

*Microlechia klimeschi* (Povolný, 1972)

*Microlechia karsholti* (Nupponen, 2010)

***Cosmardia* Povolný, 1965**

*Cosmardia moritzella* (Treitschke, 1835)

*morizella* (Geyer, 1836)

*roseella* (Zetterstedt, 1839)

***Lutilabria* Povolný, 1965 [167**]

*Lutilabria lutilabrella* (Mann, 1857)

*robustella* (Rebel, 1910)

*olympica* Huemer, 1993, subspecies

*Lutilabria volgensis* Anikin & Piskunov, 1996

*Lutilabria prolata* Junnilainen & Nupponen, 2010

***Klimeschiopsis* Povolný, 1967**

*Klimeschiopsis kiningerella* (Duponchel, 1843) [**168**]

*atralbella* (Palm, 1947)

*Klimeschiopsis discontinuella* (Rebel, 1899)

*Klimeschiopsis maritimaealpina* Nel & Varenne, 2011

*Klimeschiopsis terroris* (Hartig, 1938)

***Caryocolum* Gregor & Povolný, 1954 [169**]

*Caryocolum fischerella* (Treitschke, 1833)

*Caryocolum tischeriella* (Zeller, 1839) [**170**]

*Caryocolum alsinella* (Zeller, 1868) [**171**]

*albifrontella* (Heinemann, 1870)

*tristella* (Heinemann, 1870)

*semidecandriella* (Tutt, 1887)

*semidecandrella* (Threlfall & Stainton, 1887)

*Caryocolum viscariella* (Stainton, 1855)

*crepusculella* (Teich, 1889)

*Caryocolum albifaciella* (Heinemann, 1870)

*behenella* (Constant, 1890)

*Caryocolum vicinella* (Douglas, 1851) [**172**]

*inflatella* (Chrétien, 1901)

*albescens* (Bankes, 1909), unavailable

*suffusa* (Bankes, 1909), unavailable

*Caryocolum bosalella* (Rebel, 1936)

*Caryocolum sciurella* (Walsingham, 1908)

*rubidella* (Chrétien, 1908)

*Caryocolum amaurella* (Hering, 1924) [**173**]

*viscariae* (Schütze, 1926)

*Caryocolum crypticum* Huemer, Karsholt & Mutanen, 2014

*Caryocolum tredosella* Nel & Requena, 2017

*Caryocolum oculatella* (Thomann, 1930)

*ochraceella* (Thomann, 1929), homonym

*Caryocolum leucofasciatum* Huemer, 1989

*Caryocolum petryi* (Hofmann, 1899)

*rougemonti* (Rebel, 1907)

*repentella* (Chrétien, 1908)

*benanderi* (Hering, 1933)

*Caryocolum baischi* Huemer & Karsholt, 2010

*Caryocolum repentis* Huemer & Luquet, 1992

*repentella* auct.

*Caryocolum siculum* Bella, 2008

*Caryocolum inflativorella* (Klimesch, 1938)

*xuthella* (Rebel, 1941)

*census* (Gozmány, 1954)

*Caryocolum saginella* (Zeller, 1868) [**174**]

*coussonella* (Chrétien, 1908)

*Caryocolum cauligenella* (Schmid, 1863) [**175**]

*Caryocolum trauniella* (Zeller, 1868)

*Caryocolum peregrinella* (Herrich-Schäffer, 1854) [**176**]

*melantypella* (Mann, 1877)

*Caryocolum delphinatella* (Constant, 1890)

*fiorii* (Klimesch, 1953)

*Caryocolum provinciella* (Stainton, 1869)

*Caryocolum mucronatella* (Chrétien, 1900)

*poschiavensis* (Rebel, 1936)

*Caryocolum leucomelanella* (Zeller, 1839) [**177**]

*gypsophilae* (Stainton, 1869)

*Caryocolum mazeli* Huemer & Nel, 2005

*Caryocolum leucothoracellum* (Klimesch, 1953)

*Caryocolum schleichi* (Christoph, 1872) [**178**]

*syriacum* Povolný, 1977

*dianthella* (Chrétien, 1925), subspecies

*hackeri* Derra, 1985

*improvisella* (Rebel, 1936), subspecies

*Caryocolum arenariella* (Benander, 1937) [**178**]

*Caryocolum marmorea* (Haworth, 1828) [**179**]

*manniella* (Zeller, 1839)

*marmorella* (Doubleday, 1859), emendation

*pulchra* (Wollaston, 1858), subspecies

*mediocorsa* Varenne & Nel, 2013, subspecies

*marmoreum* auct.

*Caryocolum pullatella* (Tengström, 1848) [**180**]

*pulla* (Tengström, 1848)

*subtractella* (Walker, 1864)

*livoniella* (Teich, 1898)

*agricolaris* (Meyrick, 1933)

*Caryocolum stramentella* (Rebel, 1935)

*emarginatum* Huemer, 1988

*Caryocolum hispanicum* Huemer, 1988

*Caryocolum confluens* Huemer, 1988

*Caryocolum srnkai* Huemer & Karsholt, 2011

*Caryocolum gallagenellum* Huemer, 1989

*Caryocolum fraternella* (Douglas, 1851)

*intermediella* (Hodgkinson, 1897)

*Caryocolum klosi* (Rebel, 1917) [**181**]

*Caryocolum interalbicella* (Herrich-Schäffer, 1854)

*quadrella* (Fabricius, 1794), homonym

*Caryocolum laceratella* (Zeller, 1868)

*thurneri* (Pinker, 1953)

*Caryocolum dauphini* Grange & Nel, 2012

*Caryocolum blandella* (Douglas, 1852) **nom. protectum [182**]

*signatella* (Eversmann, 1844) **nom. oblitum**

*maculea* (Haworth, 1828), (*nec* Fabricius, 1794), emendation, misident.

*Caryocolum blandelloides* Karsholt, 1981

*Caryocolum horoscopa* (Meyrick, 1926) **stat. rev. [183**]

*Caryocolum jaspidella* (Chrétien, 1908)

*Caryocolum proxima* (Haworth, 1828)

*maculiferella* (Douglas, 1851)

*maculivicinella* (Bruand d´Uzelle, 1859)

*horticolla* (Peyerimhoff, 1871)

*proximum* auct.

*Caryocolum blandulella* (Tutt, 1887)

*Caryocolum arenbergeri* Huemer, 1989

*Caryocolum tricolorella* (Haworth, 1812)

*contigua* (Haworth, 1828)

*acernella* (Herrich-Schäffer, 1854)

*Caryocolum fibigerium* Huemer, 1988 [**184**]

*Caryocolum junctella* (Douglas, 1851) [**185**]

*aganocarpa* (Meyrick, 1935)

*Caryocolum cassella* (Walker, 1864)

*melanotephrella* (Erschoff, 1877)

*albifasciella* (Toll, 1936)

*subvicinella* (Hackman, 1946)

*falellum* Piskunov, 1975

*Caryocolum moehringiae* (Klimesch, 1954)

*Caryocolum petrophila* (Preissecker, 1914)

*kemnerella* (Palm, 1947)

*Caryocolum huebneri* (Haworth, 1828)

*hubnerella* (Doubleday, 1866)

*knaggsiella* (Stainton, 1866)

*Caryocolum kroesmanniella* (Herrich-Schäffer, 1854)

*huebneri* auct.

***Tila* Povolný, 1965**

*Tila capsophilella* (Chrétien, 1900)

***Pogochaetia* Staudinger, 1879**

*Pogonochaetia* Rye, 1881

*Chaetopogon* Rye, 1881

*Pogochaetia solitaria* Staudinger, 1879

*ocymoidella* (Walsingham, 1900), subspecies

*cabreretsi* Povolný, 1981

***Agonochaetia* Povolný, 1967**

*Sautereopsis* Povolný, 1965

*Agonochaetia terrestrella* (Zeller, 1872) [**186**]

*muestairella* (Müller-Rutz, 1922)

*Agonochaetia intermedia* Sattler, 1968

*Agonochaetia quartana* Povolný, 1990

***Canarischema* Karsholt, 2017**

*Canarischema fuerteventura* Karsholt, 2017

***Sattleria* Povolný, 1965 [187**]

*Sattleria melaleucella* (Constant, 1865) [**188**]

*mariae* (Frey, 1867), unavailable

*fusca* (Burmann, 1954)

*Sattleria arcuata* Pitkin & Sattler, 1991

*Sattleria pyrenaica* (Petry, 1904) [**189**]

*Sattleria taurandi* Nel & Varenne, 2019

*Sattleria karsholti* Huemer & Hebert, 2011

*Sattleria cottiella* Huemer & Hebert, 2011

*Sattleria marguareisi* Huemer & Sattler, 1992

*Sattleria izoardi* Huemer & Sattler, 1992

*Sattleria graiaeella* Huemer & Hebert, 2011

*Sattleria dolomitica* Huemer, 2014

*Sattleria basistrigella* Huemer, 1997

*Sattleria triglavica* Povolný, 1987

*Sattleria basistrigella* Huemer, 1997

*basistrigella* (Müller-Rutz, 1934), unavailable

*Sattleria dinarica* Huemer, 2014

*Sattleria haemusi* Huemer, 2014

*Sattleria dzieduszyckii* (Nowicki, 1864)

*tatrica* (Gregor & Povolný, 1955)

*Sattleria angustispina* Pitkin & Sattler, 1991

*Sattleria breviramus* Pitkin & Sattler, 1991

*Sattleria sophiae* Timossi, 2014

*Sattleria styriaca* Pitkin & Sattler, 1991

**LitiniBruand d’Uzelle 1859 [190**]

Teleiodini Piskunov, 1973

Exoteleiini Omelko, 1999

***Schneidereria* Weber, 1957**

*Schneidereria pistaciella* Weber, 1957 [**191**]

***Teleiodes* Sattler, 1960**

*Dubitationis* Omelko & Omelko, 1998

*Teleia* Heinemann, 1870, homonym

*Teleiodes vulgella* ([Denis & Schiffermüller], 1775) [**191**]

*aspera* (Haworth, 1828)

*Teleiodes italica* Huemer, 1992 [**192**]

*gallica* Huemer, 1992

*Teleiodes brevivalva* Huemer, 1992 [**192**]

*Teleiodes wagae* (Nowicki, 1860)

*marsata* Piskunov, 1973

*Teleiodes saltuum* (Zeller, 1878) [**193**]

*nigristrigella* (Wocke, 1898)

*Teleiodes kaitilai* Junnilainen, 2010 [**193**]

*Teleiodes luculella* (Hübner, 1813) [**194**]

*subrosea* (Haworth, 1828)

*Teleiodes flavimaculella* (Herrich-Schäffer, 1854) [**195**]

*rufipunctella* (Steudel, 1882)

*dealbella* (Klemensiewicz, 1902), unavailable

*herrichi* (Dufrane, 1955), unavailable

*Teleiodes albidorsella* Huemer & Karsholt, 1999

*Teleiodes albiluculella* Huemer & Karsholt, 2001

***Neotelphusa* Janse, 1958**

*Neotelphusa sequax* (Haworth, 1828)

*apicistrigella* (Duponchel, 1843)

*sequaxella* (Bruand d´Uzelle, 1859)

*sequacella* (Doubleday, 1859), emendation

*Neotelphusa huemeri* (Nel, 1998)

*pseudocisti* Leraut, 1997, unavailable

*Neotelphusa traugotti* (Huemer & Karsholt, 2001)

*Neotelphusa cisti* (Stainton, 1869)

***Carpatolechia* Căpuşe, 1964**

*Vicina* Omelko, 1999

*Carpatolechia decorella* (Haworth, 1812)

*humeralis* (Zeller, 1839)

*lyellella* (Humphreys & Westwood, 1845)

*incretella* (Duponchel, 1845)

*humeralella* (Bruand d´Uzelle, 1851), emendation

*marmoripennella* (Bruand d´Uzelle, 1851)

*pisticella* (Nowicki, 1860)

*scabra* (Staudinger, 1870)

*erschoffii* (Frey, 1880)

*subericolella* (Caradja, 1920), unavailable

*buckwelli* (Lucas, 1956)

*dumitrescui* Căpuşe, 1964

*Carpatolechia aenigma* (Sattler, 1983)

*Carpatolechia fugitivella* (Zeller, 1839)

*vovkella* (Piskunov, 1973)

*melanella* (Romaniszyn, 1933), unavailable

*Carpatolechia fugacella* (Zeller, 1839)

*nigrofasciella* (Bruand d´Uzelle, 1851)

*Carpatolechia minor* (Kasy, 1978)

*Carpatolechia filipjevi* (Lvovsky & Piskunov, 1993)

*Carpatolechia alburnella* (Zeller, 1839)

*seniculella* (Eversmann, 1844)

*radiella* (Krulikowsky, 1909), unavailable

*Carpatolechia notatella* (Hübner, 1813)

*euratella* (Herrich-Schäffer, 1854)

*oskella* (Piskunov, 1973)

*Carpatolechia proximella* (Hübner, 1796)

*peritella* (Constant, 1885)

*ochracella* (Romaniszyn, 1933), unavailable

*Carpatolechia intermediella* Huemer & Karsholt, 1999

*Carpatolechia epomidella* (Tengström, 1869)

***Pseudotelphusa* Janse, 1958**

*Sattleria* Căpuşe, 1968, homonym

*Klaussattleria* Căpuşe, 1968

*Pseudotelphusa scalella* (Scopoli, 1763) [**196**]

*aleella* (Fabricius, 1794)

*bicolorella* (Treitschke, 1832)

*Pseudotelphusa istrella* (Mann, 1866)

*decuriella* (Mann, 1872)

*trifasciella* (Rebel, 1916)

*Pseudotelphusa occidentella* Huemer & Karsholt, 1999

*Pseudotelphusa paripunctella* (Thunberg, 1794)

*tigratella* (Costa, 1834)

*triparella* (Zeller, 1839)

*trijugella* (Erschoff, 1877)

*sultanella* (Caradja, 1920)

*griseella* (Preissecker, 1931), unavailable

*myricae* (Gilles, 1936), unavailable

*pseudowagae* (Svensson, 1993), unavailable

*Pseudotelphusa tessella* (Linnaeus, 1758)

*albinigrella* ([Denis & Schiffermüller], 1775)

*sturmella* (Hübner, 1825)

*berberidella* (Hübner, 1825)

*funestella* (Geyer, 1832)

*alboquadrella* (Bruand d´Uzelle, 1859)

***Istrianis* Meyrick, 1918**

*Pseudoteleia* Amsel, 1935

*Istrianis myricariella* (Frey, 1870)

*Istrianis arenicolella* (Caradja, 1920)

*amilcarella* (Lucas, 1933)

*Istrianis pseudomyricariella* Bidzilya & Karsholt, 2015

*Istrianis nilssoni* Bidzilya & Karsholt, 2015

*Istrianis brucinella* (Mann, 1872)

*Istrianis femoralis* (Staudinger, 1876)

*comedonella* (Staudinger, 1879)

*gravosensis* (Rebel, 1937)

*angustipennis* (Rebel, 1941)

*funebrella* (Rebel, 1941)

*squamodorella* auct.

*Istrianis piskunovi* Bidzilya & Karsholt, 2015

***Streyella* Janse, 1958**

*Streyella canariensis* (Walsingham, 1908)

*Streyella anguinella* (Herrich-Schäffer, 1861)

*ostentella* (Zerny, 1934)

***Teleiopsis* Sattler, 1960**

*Teleiopsis terebinthinella* (Herrich-Schäffer, 1856)

*Teleiopsis latisacculus* Pitkin, 1988

*Teleiopsis diffinis* (Haworth, 1828) [**197**]

*dissimilella* (Treitschke, 1833)

*scabidella* (Zeller, 1839)

*friesella* (Zetterstedt, 1839)

*diffinella* (Doubleday, 1859), emendation

*groenliensis* (Strand, 1920), unavailable

*Teleiopsis lunariella* (Walsingham, 1908)

*Teleiopsis bagriotella* (Duponchel, 1840) [**197**]

*elatella* (Herrich-Schäffer, 1854)

*Teleiopsis laetitiae* Schmid, 2011 [**197**]

*Teleiopsis lindae* Schmid, 2011

*Teleiopsis albifemorella* (Hofmann, 1867) [**197**]

*Teleiopsis paulheberti* Huemer & Mutanen, 2012 [**197**]

*Teleiopsis rosalbella* (Fologne, 1862) [**197**]

***Xenolechia* Meyrick, 1895 [198**]

*Xenolechia aethiops* (Humphreys & Westwood, 1845)

*aterrima* (Edleston, 1844)

*aethiopella* (Doubleday, 1859), emendation

*squamulella* (Peyerimhoff, 1871)

*tristis* (Staudinger, 1879)

*Xenolechia lindae* Huemer & Karsholt, 1999

*Xenolechia pseudovulgella* Huemer & Karsholt, 1999

***Altenia* Sattler, 1960**

*Altenia perspersella* (Wocke, 1862)

*empetrella* (Karvonen, 1932)

*Altenia scriptella* (Hübner, 1796) [**199**]

*Altenia elsneriella* Huemer & Karsholt, 1999

*Altenia mersinella* (Staudinger, 1879)

*melanostictella* (Ragonot, 1895)

*sagittella* (Caradja, 1920)

*praedicta* (Meyrick, 1923)

*tribolopis* (Meyrick, 1927)

*Altenia wagneriella* (Rebel, 1926)

*danilevskyi* (Piskunov, 1973)

*Altenia modesta* (Danilevsky, 1955)

***Recurvaria* Haworth, 1828**

*Lita* Kollar, 1832

*Telea* Stephens, 1834, homonym

*Aphanaula* Meyrick, 1895

*Hinnebergia* Spuler, 1910

*Recurvaria nanella* ([Denis & Schiffermüller], 1775)

*pumilella* ([Denis & Schiffermüller], 1775)

*nana* Haworth, 1828, emendation

*crataegella* Busck, 1903

*unicolor* Rebel, 1927

*pruniella* auct.

*Recurvaria leucatella* (Clerck, 1759)

*leucatea* Haworth, 1828, emendation

*albocingulella* (Duponchel, 1839)

*Recurvaria thomeriella* (Chrétien, 1901)

*Recurvaria costimaculella* Huemer & Karsholt, 2001

***Coleotechnites* Chambers, 1880**

*Evagora* Clemens, 1860, homonym

*Eidothea* Chambers, 1873 (emendation and homonym)

*Eucordylea* Dietz, 1900

*Pulicalvaria* Freeman, 1963

*Coleotechnites piceaella* (Kearfott, 1903)

*nigra* (Kearfott, 1903), homonym

*obscurella* (Kearfott, 1907)

***Exoteleia* Wallengren, 1881**

*Paralechia* Busck, 1903

*Heringia* Spuler, 1910, homonym

*Heringiola* Strand, 1917

*Exoteleia dodecella* (Linnaeus, 1758) [**200**]

*duodecimcristata* (Retzius, 1783), unavailable

*punctulata* (Fourcroy, 1785)

*dodecea* (Haworth, 1828), emendation

*annulicornis* (Stephens, 1834)

*favillaticella* (Zeller, 1839)

*reussiella* (Ratzeburg, 1840)

*Exoteleia succinctella* (Zeller, 1872)

*oribatella* (Rebel, 1918)

***Stenolechia* Meyrick, 1894**

*Poecilia* Heinemann, 1870, homonym

*Gibbosa* Omelko, 1988

*Stenolechia gemmella* (Linnaeus, 1758)

*nivella* (Fabricius, 1794)

*nivea* (Haworth, 1828), emendation

*lepidella* (Zeller, 1839)

*nigrovittella* (Duponchel, 1839)

***Parastenolechia* Kanazawa, 1985**

*Origo* Omelko, 1988

*Tutor* Omelko, 1988

*Laris* Omelko, 1988

*Parastenolechia nigrinotella* (Zeller, 1847)

*nigralbella* (Herrich-Schäffer, 1854), unavailable

***Stenolechiodes* Elsner, 1996**

*Stenolechiodes pseudogemmellus* Elsner, 1996

*Stenolechiodes macrolepiellus* Huemer & Karsholt, 1999

***Parachronistis* Meyrick, 1925**

*Cochlevalva* Omelko, 1986

*Dentivalva* Omelko, 1986

*Parachronistis albiceps* (Zeller, 1839) [**201**]

*albicipitella* (Herrich-Schäffer, 1854), emendation

*albicapitella* (Doubleday, 1859), emendation

***Schistophila* Chrétien, 1899**

*Schistophila laurocistella* Chrétien, 1899

*striatana* (Lucas, 1937)

**Unplaced genus**

“*Telphusa*” *cistiflorella* (Constant, 1890) [**202**]

## Comments on the checklist

Approximately 200 comments on systematic problems, taxonomic changes and particularly potential cryptic diversity, are mainly derived from molecular data and are cross-referenced in the checklist: [1] – [202].

[1] Anacampsidae Bruand d’Uzelle, 1851 has priority over Gelechiidae Stainton 1854. The former name has hardly been used (Sattler 1973) and the use of the older synonym would threaten stability. Following Art. 23.9.3 (ICZN) the case should therefore be referred to the Commission for a ruling under the plenary power. The year of description of Anacampsidae is according to Viette (1977).

[2] *Stomopteryx*. This genus is in need of a taxonomic revision and includes several probable cases of cryptic diversity, and equally probably cases of over-splitting.

[3] *Stomopteryx nugatricella* / *S. mongolica* / *S. lineolella.* The taxonomy of these species is unresolved and should be checked in upcoming revisionary work. Junnilainen et al. (2010) separated *S. mongolica* and *S. lineolella* on morphological differences they observed in male genitalia but at the same time stated that European specimens of *S. mongolica* differ from typical Mongolian vouchers (Note: They did not compare *S. mongolica* from southern Russia with the externally similar *S. nugatricella* from Spain). DNA barcodes do not support species status of all these taxa which cluster with very low divergences in the same BIN. We therefore believe that taxonomic over-splitting cannot be excluded and would be a reasonable explanation for the current species concept, although barcode sharing between some taxa cannot be excluded.

[4] *Stomopteryx deverrae*. We have barcoded only North African specimens so far, including a syntype from Algeria, and the material from Spain should be sequenced in future to prove the occurrence in Europe.

[5] *Stomopterayx flavoclavella*. European samples from Spain slightly differ from a sequenced syntype from Morocco and cluster in a separate BIN. The suspected conspecificity will be addressed in an upcoming revision.

[6] *Stomopteryx remissella*. This species represents an unresolved species complex. DNA barcodes show an extraordinarily high and largely geographic variation, reflected by eight different BINs and differences in phenotype. The recently described *Stomopteryx spathulella* (Nel et al. 2019) probably belongs to one of the BINs summarized for *S. remissella*. However, the whole complex requires thorough revisionary work and a re-evaluation of available names.

[7] *Stomopteryx flavipalpella*. A genetically variable species which clusters into three BINs without obvious geographic variation.

[8] *Aproaerema*. Recently Aaarvik et al. (2017) synonymized the widely accepted and diverse genus *Syncopacma* with *Aproaerema*, resulting in numerous nomenclatural changes. We here propose the following new or revised combinations: *Aproaerema incognitana* (Gozmány, 1957) comb. nov., *Aproaerema cinctelloides* (Nel & Varenne, 2012) comb. nov., *Aproaerema wormiella* (Wolff, 1958) comb. nov., *Aproaerema azosterella* (Herrich-Schäffer, 1854) comb. nov., *Aproaerema montanata* (Gozmány, 1957) comb. nov., *Aproaerema cincticulella* (Bruand, 1851) comb. nov., *Aproaerema buvati* (Nel, 1995) comb. nov., *Aproaerema linella* (Chrétien, 1904) comb. nov., *Aproaerema captivella* (Herrich-Schäffer, 1854) comb. nov., *Aproaerema semicostella* (Staudinger, 1871) comb. nov., *Aproaerema steppicola* (Junnilainen, 2010) comb. nov., *Aproaerema cottienella* (Nel, 2012) comb. nov., *Aproaerema genistae* (Walsingham, 1908) comb. rev., *Aproaerema thaumalea* (Walsingham, 1905) comb. rev. The genus *Aproaerema* includes several yet unresolved DNA barcode clusters which may partly reflect cryptic diversity and therefore requires revisionary work.

[9] *Aproaerema cinctella*. This species clusters into two weakly separated DNA barcode clusters with max. distance of 1.86%, probably reflecting intraspecific variation.

[10] *Aproaerema linella*. A unicolorous, dissected male from Montenegro largely corresponds with the lectotype figured by Nel et al. (1996) in the male genitalia. However, the original description of *A. linella* as well as bred samples from the type area characterize *S. linella* as a species with a distinct yellow-orange subterminal fascia or costal and tornal spots and a further yellow spot in the middle of the forewing. A female from northern Italy clustering in a separate BIN matches these phenotypical characters better and also largely agrees in the genitalia. However, in the absence of molecular data from the type-locality, identification of both specimens remains uncertain and we only tentatively assign the name *A. linella* to the former specimen and leave the latter as an unidentified cluster.

[11] *Aproaerema suecicella*. Two strongly divergent BINs (4.33% min. distance) show a geographic pattern and need to be tested for potential cryptic diversity.

[12] *Aproaerema karvoneni*. Three weakly separated BINs (1.61% min. distance) partially show geographic (probably intraspecific) variation.

[13] *Aproaerema anthyllidella*. The moderate DNA barcode variation with three BINs may reflect cryptic diversity, as e.g., suspected for the current synonym *A. natrixella* (Schmid pers. comm.) and some of the other five current synonyms, but has to be carefully checked with an integrative taxonomic approach.

[14] *Iwaruna*. Species in this genus share their BINs and partially overlap in DNA barcodes (*I. biguttella* and *I. klimeschi*) but differ in morphology. DNA barcodes of *I. heringi*, a species requiring taxonomic re-assessment, are unknown.

[15] *Anacampsis populella* / *A. blattariella*. A population from western Austria (Vorarlberg) shares its BIN with a unique specimen of *A. populella* from Finland though matching *A. blattariella* in morphology. This is most likely a case of a so far unrecognized introgression in these two species, though the weakly deviating DNA barcode may require further studies. All other sequenced specimens of both species group in separate BINs.

[16] *Anacampsis scintillella*. Two specimens from Spain cluster in a separate BIN.

[17] *Anacampsis obscurella*. Our limited data indicates geographically separated species with three BINs but requires additional revisionary work.

[18] *Mesophleps*. The genus was recently revised by Li and Sattler (2012). Two strongly deviating DNA barcode clusters (and BINs) from Spain and Greece probably represent undescribed species.

[19] *Nothris*. The sequence of species follows the revision by Karsholt and Šumpich (2015).

[20] *Nothris gregerseni*. A specimen from Sweden clusters into a unique BIN (3.83% min. distance, but probably representing only an intraspecific split.

[21] *Nothris radiata*. The yet unpublished occurrence in Europe is based on a DNA barcoded specimen from Macedonia (Šumpich in prep.).

[22] *Neofaculta ericetella*. This species shows high intraspecific DNA barcode variation and clusters into three BINs without geographic variation.

[23] *Neofaculta taigana*. The occurrence of this Asian species in Europe will be dealt with separately by Aarvik, Berggren, Karsholt and Mutanen.

[24] *Hypatima rhomboidella*. Genetically variable species clustering into three BINs without geographic variation.

[25] *Anarsia*. The genus requires revisionary work and probably includes two undescribed species from Greece and Cyprus respectively.

[26] *Anarsia bilbainella*. A unique sequence from the type-area in Spain clusters into a separate BIN (1.26% min. distance).

[27] *Dichomeris*. As currently understood, *Dichomeris* is the largest genus within the Gelechiidae. Ponomarenko (2009) lists 582 species (+ some species placed in *Acanthophila* and *Uliaria*). Due to the high external diversity, many genera were erected, especially for tropical species. Ponomarenko (2009) and Vives Moreno (2014) listed more than 80 synonyms of *Dichomeris*. Here we only consider genera relevant for the European fauna. The genus includes one probably undescribed species from Spain.

[28] *Dichomeris limbipunctellus / D. neatodes*. These two taxa, which have been regarded as conspecific, differ in phenotypy, show a different distribution pattern in the eastern (*D. neatodes*) and western Mediterranean (*D. limbipunctellus*), and cluster into two BINs. We accordingly list them as separate species and reinstate *D. neatodes* sp. rev. as a valid species.

[29] *Dichomeris juniperella*. The species splits into two strongly divergent BINs (5.26% min. distance), one widespread, and the other restricted to the southern Alps, reflecting possible cryptic diversity.

[30] *Dichomeris rasilella*. A single DNA barcode from Russia is highly divergent from other samples and clusters into a separate BIN (6.26% min. distance).

[31] *Acompsia*. The sequence of species follows the revision by Huemer and Karsholt (2002).

[32] *Acompsia pyrenaeella*. The species clusters into three BINs, one shared with phenotypically compared specimens of *A. tripunctella* and *A. antirrhinella*, indicating occasional introgression.

[33] *Acompsia antirrhinella*. Despite diagnostic morphological characters, this species shares the only known BIN with two genetically variable species, *A. pyrenaella* and *A. tripunctella*. See also comments under these species.

[34] *Acompsia maculosella*. Sequences of specimens from the southern Alps group into a separate BIN and are also separated by reduced forewing markings, but agree in genitalia morphology and are therefore tentatively considered as *A. maculosella*. [

[35] *Acompsia tripunctella*. A genetically highly variable species, which clusters into seven BINs, possibly reflecting cryptic diversity requiring revisionary work. One BIN is shared with *A. pyrenaeella* and *A. antirrhinella*. See also comments under these species.

[36] *Brachmia*. A species from Greece (Crete) is probably undescribed (Berggren in prep.).

[37] *Brachmia dimidiella*. A genetically variable species clustering into three different BINs.

[38] *Helcystogramma lamprostoma*. Male and female genitalia match *Helcystogramma* Zeller, 1877 and the species was placed in this genus in recent papers (Agassiz and Bidzilya 2016, Bidzilya et al. 2019, Karsholt and Huemer 2017). The DNA barcode indicates the species as sister-group of the other European *Helcystogramma*.

[39] *Pseudosophronia*. The identity of the three currently listed European species is somewhat doubtful and requires further analysis. Corley (2001) gives a clear indication that alleged diagnostic characters for *P. constanti* described by Nel (1998) in fact fall within the intraspecific variation of *S. exustellus*. Furthermore, a successfully sequenced specimen from the type-area of *P. constanti* fully agrees with *P. exustellus* from France and Spain. We therefore formally synonymize *P. constanti* with *P. exustellus* (syn. nov.).

[40] *Pexicopia malvella*. The species splits into two BINs (4.33% min. distance) without geographic distinction and requires further analysis. The geographic variation in the forewing colour and pattern between specimens from Central Europe and South Europe is not reflected in the DNA barcode.

[41] Apatetrini. Genera and species of this tribe are in need of revision. Several of the included taxa do not cluster together in a barcode-based NJ tree and Apatetrini sensu auct. is likely not a monophyum.

[42] *Dactylotula kinkerella*. The species splits into two divergent clusters representing two BINs (4.49% min. distance).

[43] *Apatetris*. The two species listed here, *A. agenjoi* and *A. mediterranella*, are based on morphology not strictly congeneric with the type of the genus (*A. mirabella* Staudinger, 1879 from Turkey) and probably also not with each other, but are left here pending forthcoming revisionary work. Similarly, two yet unidentified species which are probably undescribed are not closely related and only tentatively assigned to *Apatetris*.

[44] *Apatetris mediterranella*. The species clusters into two geographically separate BINs (3.05% min. distance) and requires further revision.

[45] *Catatinagma trivittellum*. The species splits into two geographically separate and strongly divergent clusters, representing two BINs (5.11% min. distance). These should be tested for potential cryptic diversity with further sampling and a comprehensive morphological analysis.

[46] *Catatinagma kraterella*. The species does not cluster close to the type of the genus (*C. trivittellum*), instead appears closer to *Apatetris mediterranella*. It is, however, left in *Catatinagma* pending discovery of the unknown female and forthcoming revisionary work.

[47] *Chrysoesthia*. This genus lacks generic revision. Three yet unassigned, but sequenced species, may partly belong to the insufficiently revised taxa of the European fauna.

[48] *Chrysoesthia drurella*. This species splits into two strongly divergent BINs (3.69% min. distance) which partly overlap geographically and require careful re-assessment.

[49] *Chrysoesthia atriplicella* / *C. gaditella* / *C. aletris*. Morphological revisionary work and additional DNA barcoding efforts are required to determine if these three names represent one or more species. *C. halymella* (Amsel, 1935) also belongs to this complex (Bidzilya et al. 2019).

[50] *Metanarsia modesta*. The species splits into two BINs, one only known from extra-European Armenia.

[51] *Oecocecis guyonella*. We were able to dissect both sexes from specimens provided by Christian Gibeaux. The female genitalia are rather similar to *Metanarsia*, but the male genitalia are strongly different. Therefore, and in the absence of molecular data, the systematic position is tentative and requires further revisionary work.

[52] For a discussion of the validity of Palumbininae, see Ponomarenko (2005, 2008b) and Karsholt et al. (2013).

[53] *Bryotropha*. The sequence of species follows the revision by Karsholt and Rutten (2005). We did not obtain DNA barcodes from the taxa listed in that publication as ‘*Bryotropha* species A’ and ‘*Bryotropha* species B’.

[54] *Bryotropha terrella*. Two deviating DNA barcodes from Austria group into a separate BIN (2.94% min. distance) and the corresponding specimens require careful re-evaluation.

[55] *Bryotropha desertella*. A genetically variable species clustering into three BINs without geographical structure.

[56] *Bryotropha hulli*. The species clusters into two BINs without geographical separation.

[57] *Bryotropha affinis*. This species shares its BIN with one BIN of *B. umbrosella*.

[58] *Bryotropha umbrosella*. The species clusters into two BINs, one shared with *B. affinis*, which differs in phenotype and genitalia morphology (Karsholt and Rutten 2005).

[59] *Epidola*. Unrevised genus. The identity of *Epidola grisea*, described from a single male without an abdomen and collected in Sardinia (Amsel 1942) remains obscure and needs further revisionary work. We therefore do not include it in the current checklist of European Gelechiidae.

[60] *Epidola semitica*. This species was described from a single male from Israel, but according to recently collected material it also occurs in Greece (new record for Europe, for detailed data see dataset in BOLD).

[61] *Aristotelia*. This genus is in strong need of a generic revision and includes several probably undescribed species.

[62] *Aristotelia decurtella*. This species genetically clusters into two BINs (2.25% min. distance) which are in need of morphological revision.

[63] *Aristotelia ericinella*. Specimens from Sardinia cluster separately into a different BIN (2.73% min. distance) and are considered as a separate species.

[64] *Aristotelia subdecurtella*. Two barcode clusters, grouping into different BINs that overlap in distribution.

[65] *Aristotelia subericinella*. The species identity is based on barcoded material from the type area (eastern Austria). Several additional clusters formerly identified as *A. subericinella* probably include cryptic diversity and are in strong need of taxonomic revision. These clusters are considered as unidentified taxa in our analysis.

[66] *Aristotelia billii*. DNA barcodes of this species are based upon the successfully sequenced holotype and prove a wide distribution from the Mediterranean to Kirgizia.

[67] *Caulastrocecis*. The genus is in need of revision.

[68] *Caulastrocecis furfurella* / *C. cryptoxena*. The former was considered as a senior synonym of *C. cryptoxena* but both are clearly divergent in DNA barcodes and represent different species (Bidzilya and Karsholt in prep.). We therefore reinstate *C. cryptoxena* sp. rev. as a valid species.

[69] *Paranarsia*. The systematic position of this genus is not fully resolved. The genitalia somewhat resemble those of *Caulastrocesis* but DNA barcodes are distant. Here we follow Elsner et al. (1999) in placing these two genera next to each other.

[70] *Megacraspedus*. This genus was recently revised with 27 newly described species from Europe (Huemer and Karsholt 2018). The authors recognized extraordinary intraspecific DNA barcode variation within several species, some of which might include additional cryptic diversity.

[71] *Megacraspedus lanceolellus*. Genetically extremely variable species, which clusters into 19 BINs of mainly geographic variation, with an intraspecific DNA barcode variation of 12.5% (Huemer and Karsholt 2018).

[72] *Megacraspedus dolosellus*. Genetically extremely variable species, which clusters into 23 BINs of mainly geographic variation, with an intraspecific DNA barcode variation of 13.8% (Huemer and Karsholt 2018).

[73] *Megacraspedus spinophallus*. Two barcode clusters, representing separate BINs with records from nearby localities.

[74] *Megacraspedus binotella*. Genetically variable species, which clusters into three BINs without clear geographic separation.

[75] *Megacraspedus brachypteris*. Genetically variable species, which clusters into four BINs without clear geographic separation.

[76] *Megacraspedus andreneli*. Two barcode clusters, representing separate BINs with records from nearby localities.

[77] *Megacraspedus imparellus*. Genetically variable species, which clusters into three BINs with probable geographic separation.

[78] *Megacraspedus teriolensis*. Genetically variable species, which clusters into two geographically distinct BINs.

[79] *Dirhinosia*. Species in this genus partly share DNA barcodes (*D. cervinella* and *D. interposita*) but differ in morphology (Bidzilya and Budashkin 2015).

[80] *Psamathocrita*. The genus is in need of revision. A probably undescribed species has been studied by Tokár and Junnilainen (in litt.) and Barton (in litt.).

[81] *Ivanauskiella*. This small genus seems to be more diverse than hitherto recognized, reflected an unidentified species from Russia and Spain. Some of the species are found in association with *Limonium* which is a likely host plant of the larvae (OK unpublished). *Spatuncusella* Nel & Varenne, 2013 was recently synonymized with *Ivanauskiella* (Nel and Varenne 2017a).

[82] *Ivanauskiella occitanica*. This species was synonymized with *I. psamathias* by Nel and Varenne (2017a). However, it clearly differs in DNA barcodes and furthermore the forewing pattern and male genitalia figures in the original description show diagnostic characters which support a separate species. We therefore reinstate *I. occitanica* sp. rev. as a valid species.

[83] *Ptocheuusa*. The genus is in strong need of revision. Barcoding efforts for several validly described species failed to produce any sequences.

[84] *Ptocheuusa paupella*. The species clusters into three separate BINs without geographic separation.

[85] *Ptocheuusa inopella*. Two barcode clusters from Spain and Sweden represent three separate BINs and need to be re-examined.

[86] *Ptocheuusa cinerella*. We transfer *Recurvaria cinerella* Chrétien from Gelechiinae: Litini to Anomologinae as *Ptocheuusa cinerella* (Chrétien, 1908) comb. nov. The male genitalia are similar overall to other species of *Ptocheuusa* and seem sufficient for this new combination despite the lack of molecular data.

[87] *Amblypalpis*. The systematic position of this genus needs re-evaluation. It was recently published as new to Europe (Vives Moreno 2019).

[88] *Parapodia*. Material from the western and eastern Mediterranean cluster into two strongly divergent BINs (5.43% min. distance). Although an initial morphological examination of females reveals no obvious diagnostic characters, these clusters should be tested for potential cryptic diversity by examining additional material and a widened morphological approach.

[89] *Isophrictis*. Unrevised genus, which includes cases of unresolved and apparently intraspecific DNA barcode divergence and probably some undescribed species, misidentified records or unrecognized synonymies for the European fauna. So far only six out of the twelve species in the checklist have been successfully barcoded.

[90] *Isophrictis kefersteiniellus*. Genetically highly variable species, which clusters into four BINs. A thorough evaluation of this problem is necessary.

[91] *Isophrictis anthemidella*. Genetically variable species, which clusters into three BINs. A thorough evaluation of this problem is necessary.

[92] *Metzneria*. The classic generic revision by Englert (1974) is out of date and several probably undescribed species or cases of distinct (though unresolved) splits in DNA barcodes urgently require a new revisionary work.

[93] *Metzneria neuropterella*. The species clusters into two BINs (2.89% min. distance) without geographic separation.

[94] *Metzneria aestivella*. The DNA barcode of a paratype of *Metzneria expositoi* Vives, 2001 from Spain fully agrees with that of *M. aestivella*. Also, the genitalia morphology of the two taxa is virtually identical, and we therefore consider *M. expositoi* to be a synonym of *M. aestivella* (syn. nov.).

[95] *Metzneria fulva / Metzneria torosulella*. Despite distinct diagnostic characters in phenotypic appearance and in the male genitalia, both species share barcodes.

[96] *Metzneria ehikeella*. The species clusters into two BINs (2.91% min. distance) without geographic separation.

[97] *Metzneria metzneriella*. This genetically variable species splits into four partly sympatric DNA barcode clusters, representing four BINs. A careful morphological examination of the problem is advisable.

[98] *Metzneria artificella*. Two weakly separated barcode clusters, representing geographically distinct BINs (1.46% min. distance), need to be re-examined.

[99] *Metzneria aprilella*. The species splits into three geographically separated DNA barcode clusters, representing three BINs. This possible case of cryptic diversity requires careful morphological re-examination.

[100] *Metzneria subflavella*. Two DNA barcodes referring to specimens from Spain and France respectively are strongly divergent and are considered separate species. These results are supported by genitalia morphology, with the Spanish specimen likely representing an undescribed species.

[101] *Metzneria campicolella*. *Metzneria varennei* Nel, 1997 was recently shown to be a synonym of *M. campicolella* (Nel and Varenne 2017b). The generic placement of this species is tentative.

[102] *Apodia martinii*. DNA barcodes of this species and *A. bifractella* with separate BINs (6.58% min. distance) fully support the species status for this long-disputed taxon. We therefore reinstate *A. martinii* sp. rev. as a valid species. Differences from *A. bifractella* in morphology, biology and distribution still need to be studied in detail.

[103] *Pragmatodes*. This genus, which has until now been placed in Gelechiini, has always been considered monotypic and endemic to the Canary Islands. However, a group of closely related species placed under *Monochroa*, i.e., *Pragmatodes melagonella* (Constant, 1895) comb. nov., *Pragmatodes albagonella* (Varenne & Nel, 2010) comb. nov., *Pragmatodes cyrneogonlla* (Nel & Varenne, 2012) comb. nov. and *Pragmatodes parvulata* (Gozmány, 1953) comb. nov., have similar genitalia which do not fit well with *Monochroa*, and their DNA barcodes cluster separately from that genus. Moreover, the known larvae of the above-mentioned species, as well as the type species of the genus (*P. fruticosella*) all feed on plants in the family Rubiaceae, an unusual feeding substrate for Gelechiidae. The genus includes additional, probably undescribed, species from South-East Europe and the Middle East.

[104] *Pragmatodes melagonella*. Specimens initially identified as this species from France and Bulgaria differ in the DNA barcode and also morphology and are considered as separate species. The type locality of *P. melagonella* is in France.

[105] *Monochroa*. This genus is in strong need of a generic revision and includes several probably undescribed species.

[106] *Monochroa rumicetella*. Two weakly separated BINs (2.12% min. distance) without geographic separation most probably reflect intraspecific variation.

[107] *Monochroa sepicolella / M. rectifasciella*. Elsner et al. (1999) had previously discussed a two-species hypothesis which is now fully supported by two strongly divergent DNA barcode clusters representing two BINs (6.7% min. distance). *M. sepicolella* occurs in North and Central Europe, whereas the name *Monochroa rectifasciella* (Fuchs, 1902) is currently used for the species with a more southern distribution (e.g., Pastorális et al. 2013). However, this problem is in need of a thorough revisionary work taking into account all available names for both species.

[108] *Monochroa tenebrella*. Two weakly separated BINs (1.12% min. distance) without geographic separation most probably reflect intraspecific variation.

[109] *Monochroa servella*. Two BINs (2.89% min. distance) without geographic separation most probably reflect intraspecific variation.

[110] *Monochroa lucidella*. Despite a low intraspecific divergence, this species may include cryptic diversity as indicated by the morphologically and genetically (only short sequences available) weakly deviating subspecies immaculatella from Northern Italy.

[111] *Monochroa arundinetella* / *M. suffusella*. These two morphologically separate species represent one of the few cases of barcode sharing among European Gelechiidae. The author and year of description of *M. arundinetella* follow Sattler (2009).

[112] *Monochroa nomadella*. This genetically highly variable species clusters in four different and geographically separate BINs and is in strong need of revisionary work. Junnilainen et al. (2010) recognized differences in the female genitalia between specimens collected in the Ural Mountains, Central Europe, and those figured by Elsner et al. (1999). They speculated that either material from Czechia was misidentified or that it could point to cryptic diversity. Unlike the few known females from Central Europe, specimens from South Russia are slightly brachypterous which might be a further indication of a potential species complex.

[113] *Oxypteryx*. *Eulamprotes* Bradley, 1971 with the type species *E. atrella* is shown to be a synonym of *Oxypteryx* Rebel, 1911 (Bidzilya et al. 2019), resulting in a number of new nomenclatural changes. We here propose the following new combinations: *Oxypteryx nigromaculella* (Millière, 1872) comb. nov., *Oxypteryx wilkella* (Linnaeus, 1758) comb. nov., *Oxypteryx ochricapilla* (Rebel, 1903) comb. nov., *Oxypteryx superbella* (Zeller, 1839) comb. nov., *Oxypteryx mirusella* Huemer & Karsholt, 2013 comb. nov., *Oxypteryx occidentella* Huemer & Karsholt, 2011 comb. nov., *Oxypteryx libertinella* (Zeller, 1872) comb. nov., *Oxypteryx baldizzonei* Karsholt & Huemer, 2013 comb. nov., *Oxypteryx gemerensis* Elsner, 2013 comb. nov., *Oxypteryx deserta* (Piskunov, 1990) comb. nov., *Oxypteryx unicolorella* (Duponchel, 1843) comb. nov., *Oxypteryx nigritella* (Zeller, 1847) comb. nov., *Oxypteryx plumbella* (Heinemann, 1870) comb. nov., *Oxypteryx isostacta* (Meyrick, 1926) comb. nov., *Oxypteryx helotella* (Staudinger, 1859) comb. nov., *Oxypteryx parahelotella* Nel, 1995 comb. nov., *Oxypteryx graecatella* Šumpich & Skyva, 2012 comb. nov. Despite this new taxonomic approach, the genus is in strong need of revision. DNA barcodes separate into three clades seemingly supported by some morphological characters. For example, species formerly considered to be in the *E. wilkella*-group and characterized by the blackish ground colour of the forewings with silvery or whitish markings, form a separate clade. Further, the genus has an extraordinary intraspecific barcode variation with 18 sequenced species belonging to 27 BINs, with at least three yet unidentified species.

[114] *Oxypteryx nigromaculella*. A specimen from Greece clusters into a separate BIN and may represent a different species.

[115] *Oxypteryx wilkella*. Two specimens from Italy and Hungary respectively are strongly divergent from the large bulk of *E. wilkella* DNA barcodes and cluster into a separate BIN. The taxonomic status of this cluster requires careful evaluation.

[116] *Oxypteryx baldizzonei*. Two strongly divergent DNA barcode clusters, representing three BINs, have been considered as intraspecific variation by Huemer et al. (2013).

[117] *Oxypteryx libertinella*. The geographic variation of DNA barcode clusters in this genetically highly variable species with eight BINs has been discussed by Huemer et al. (2013). Currently this variation is considered as an intraspecific divergence.

[118] *Athrips.* The sequence of species follows the generic revision by Bidzilya (2005).

[119] *Athrips rancidella*. A specimen from Greece clusters separately into a second BIN (2.86% min. distance) and is in need of taxonomic re-evaluation.

[120] *Athrips amoenella*. A genetically highly variable species, which clusters into five BINs.

[121] *Neofriseria peliella*. Two weakly separated BINs (1.44% min. distance) without clear geographical separation most probably reflect intraspecific variation.

[122] *Neofriseria hitadoella*. A strongly divergent BIN from France with 3.85% min. distance to *N. hitadoella* from Morocco is considered as a probable cryptic species, but the problem needs to be carefully revised.

[123] *Neofriseria kuznetzovae*. This species was listed by Piskunov (1987) and partially by Huemer and Karsholt (1999) under the name of *N. caucasicella* Sattler, 1960. The latter occurs only in the Caucasus and has not been found elsewhere in Europe.

[124] *Sophronia*. Unrevised genus with some doubtful taxa lacking DNA barcodes.

[125] *Sophronia semicostella*. Two DNA barcode clusters, grouped into two BINs, show no clear geographic separation.

[126] *Sophronia consanguinella*. *S. marginella* was recently shown to be a junior synonym of this species (Šumpich et al. 2019).

[127] *Sophronia grandii*. The DNA barcode of a paratype of *Sophronia ascalis* Gozmány, 1951 fully agrees with that of *S. grandii*. The two taxa are virtually identical, and we therefore consider *S. ascalis* to be a synonym of *S. grandii* (syn. nov.).

[128] *Sophronia chilonella*. A single DNA barcode from Bulgaria of a specimen similar to *S. chilonella* strongly deviates and may represent the taxonomically disputed and unrevised *S. acaudella*.

[129] *Sophronia sicariellus*. A single DNA barcode sequence of 504bp from Germany strongly deviates, although it may represent intraspecific variation.

[130] *Mirificarma*. Several species show a high genetic variation which could indicate cryptic diversity. Therefore, despite available taxonomic revisions by Pitkin (1984) and Huemer and Karsholt (1999), a re-evaluation of morphology seems advisable in some species.

[131] *Mirificarma lentiginosella*. Two DNA barcode clusters, which separate into two BINs (1.7% min. distance) without geographic separation.

[132] *Mirificarma cytisella*. A genetically variable species, separated into four BINs without geographic separation.

[133] *Mirificarma monticolella*. Two DNA barcode clusters from Italy and Bulgaria are highly divergent and separate into two BINs (4.49% min. distance).

[134] *Mirificarma burdonella*. Two DNA barcodes from France show a deep split into two BINs (5.78% min. distance) and require taxonomic re-evaluation.

[135] *Mirificarma ulicinella*. Two DNA barcode clusters from France and Portugal are highly divergent and separate into two BINs (3.37% min. distance).

[136] *Aroga velocella*. The species splits into three BINs, which show no clear geographic separation. The attribution of authorship follows Joannis (1922).

[137] *Aroga flavicomella*. A genetically variable species, which splits into four BINs.

[138] *Chionodes*. The sequence of species follows the revision by Huemer and Sattler (1995).

[139] *Chionodes luctuella*. DNA barcodes from central and northern Europe cluster into separate BINs (1.87% min. distance) which are currently not confirmed by morphology.

[140] *Chionodes fumatella*. DNA barcodes from central and northern Europe cluster into three geographically partially separated BINs and need taxonomic re-assessment.

[141] *Gelechia*. This genus includes at least one additional and probably undescribed species.

[142] *Gelechia senticetella*. DNA barcodes cluster into two geographically separate BINs with min. distances > 2% to the Nearest Neighbour, and need taxonomic re-assessment.

[143] *Gelechia obscuripennis*. This disputed taxon has recently been re-considered to be a separate species based on molecular data, morphology and biology (Huemer 2019).

[144] *Agnippe*. The genus (as *Evippe* Chambers, 1873) has traditionally been placed in the Litini. DNA barcodes of two species are not supportive of the systematic position of the genus in that tribe. We therefore follow Bidzilya and Li (2010) and Metz et al. (2019) in placing *Agnippe* as an isolated genus within the Gelechiini.

[145] *Holcophora*. The genera *Holocophora* and *Aponoaea* have been synonymized recently by Adamski and Sattler (2019), based on some similarities of the type-species. However, the systematic position within the Gelechiidae remains uncertain for the time being.

[146] *Holcophora inderskella*. The species was included in *Holcophora* by Adamski and Sattler (2019). It was described from Lake Indersky in Western Kazakhstan and is here attached to the European fauna despite a distance of ca. 10 km from the type-locality to the widely accepted natural border of the Continent, the Ural River.

[147] *Gnorimoschema herbichii*. Northern European populations of this species cluster into two BINs.

[148] *Scrobipalpa*. This extraordinary diverse genus still requires some taxonomic re-assessment, reflected by several yet unidentified barcode clusters which at least partly belong to undescribed species.

[149] *Scrobipalpa aptatella*. Records from Europe (France, Italy, former Yugoslavia) are unconfirmed (Huemer and Karsholt 2010).

[150] *Scrobipalpa amseli / S. hyssopi*. Both species clusters into the same BIN but differ in morphology of the male genitalia (Huemer and Karsholt 2010). Additional material should be checked to confirm if the holotype of *S. hyssopi* represents a specimen of *S. amseli* with deformed genitalia.

[151] *Scrobipalpa reiprichi*. Two geographically separate barcodes BINs (2.57% min distance) may reflect cryptic diversity, with altogether four potential species from preliminary morphological analysis (Wiesmair et al. 2018).

[152] *Scrobipalpa caucasica*. Only known from the Caucasus. *S. benzengensis* (Povolný, 2001) is a junior synonym (Huemer and Karsholt 2010).

[153] *Scrobipalpa pauperella*. Some externally different specimens from northern Italy are slightly divergent in their DNA barcodes and may belong to a separate species.

[154] *Scrobipalpa mercantourica*. This species clusters together with *Scrobipalpa arenbergeri* but according to the original description differs in morphology. A taxonomic re-assessment seems advisable to fix the status of the taxon.

[155] *Scrobipalpa alterna* / *S. lutea.* Both species share barcodes and are virtually indistinguishable in genitalia characters, although the ground colour of the forewings is usually distinct with rare intermediates. A re-assessment of this group is in preparation (Bidzilya in litt.).

[156] *Scrobipalpa artemisiella* / *Scrobipalpa stangei*. These two species are clearly separated by their biology and female genitalia morphology, but share one barcode BIN. A second BIN of *S. artemisiella* based on a single sequence most probably reflects intraspecific variation.

[157] *Scrobipalpa bryophiloides*. A genetically variable species which clusters into two separate BINs and requires further evaluation.

[158] *Scrobipalpa ocellatella*. DNA barcodes of this species clusters into two weakly separated BINs (1.44% min distance), most probably reflecting intraspecific variation.

[159] *Scrobipalpa salinella* / *S. salicorniae / S. spergulariella.* Although these species show diagnostic morphology (Huemer and Karsholt 2010) and (two) unique DNA barcode haplotypes, they cluster into the same BIN. The third species, viz. *S. spergulariella*, has not yet been barcoded.

[160] *Scrobipalpa halymella / S. stabilis.* Both species cluster into the same BIN but differ weakly in morphology (Huemer and Karsholt 2010).

[161] *Scrobipalpula*. All five successfully sequenced species share BINs, but still show species-specific DNA barcode haplotypes.

[162] *Keiferia lycopersicella*. An American species introduced to Europe in 2008 which apparently has not established permanent populations (Huemer and Karsholt 2010).

[163] *Ephysteris promptella*. A genetically highly variable species clustering into four BINs. A taxonomic re-evaluation of this problem is necessary.

[164] *Ephysteris diminutella*. Two strongly divergent and geographically separate DNA barcode clusters reflected by two BINs (5.94% min. distance) require taxonomic revision.

[165] *Ephysteris inustella*. The year of description follows Sattler (2011). The different interpretation by Huemer & Karsholt (2019) with *inustella* originally published in synonymy and only made available in 1847 is contradicted by the Code, Article 11.6.1. “However, if such a name published as a junior synonym had been treated before 1961 as an available name and either adopted as the name of a taxon or treated as a senior homonym, it is made available thereby but dates from its first publication as a synonym.”

[166] *Ochrodia*. An unidentified species from Greece (Crete) clusters with specimens from Saudia Arabia. The genus is in need of revision.

[167] *Lutilabria lutilabrella*. DNA barcodes from Slovenia and Slovakia cluster into separate BINs (3.41% min. distance) and need revisionary work.

[168] *Klimeschiopsis kiningerella*. Specimens from northern Italy cluster into a BIN separate from all other samples from various parts of Europe.

[169] *Caryocolum*. Despite extensive past revisionary work on this genus, it still includes a remarkable amount of unresolved taxonomic problems with several potential cryptic species.

[170] *Caryocolum tischeriella*. DNA barcodes cluster into three BINs without geographic separation.

[171] *Caryocolum alsinella*. A genetically highly variable species with strongly divergent DNA barcode clusters separated into three BINs. A thorough taxonomic re-assessment seems necessary.

[172] *Caryocolum vicinella*. DNA barcodes cluster into four BINs. A thorough taxonomic re-assessment seems necessary.

[173] *Carayocolum amaurella*. This genetically highly variable species clusters into five BINs, but alleged cryptic diversity is not supported by morphology (Huemer et al. 2014).

[174] *Caryocolum saginella*. DNA barcode sequences with two BINs (5.46% min. distance). clearly support the existence of a separate species in the SW-Alps (Huemer in prep.).

[175] *Caryocolum cauligenella*. A single specimen from Spain strongly deviates in DNA barcode with a separate BIN and *C. saginella* instead of *C. cauligenella* as Nearest Neighbor (5.46% min. distance). However, the specimen clearly matches the latter in phenotypy and needs taxonomic re-assessment.

[176] *Caryocolum peregrinella*. This species splits into three highly divergent allopatric clusters which most probably represent different species (Huemer in prep.). One of the major problems in resolving the taxonomic mismatches is the status of the holotype of *C. peregrinella*, a female without an abdomen and unknown type-locality, stated as Europe (Huemer 1988).

[177] *Caryocolum leucomelanella.* Two DNA barcode clusters with separate BINs (2.73% min. distance) show no geographic pattern.

[178] *Caryocolum schleichi / C. arenariella*. Initially described as different species, the largely allopatric taxa of this group have been merged into a single species by Huemer (1988). However, all these taxa are separated phenotypically and by characters in the male genitalia. As a consequence, Aarvik et al. (2017) give species status to the northern European population and re-introduced it as *C. arenariella*. Following an initial genetic analysis of the group (Huemer et al. 2014) this taxonomic change seems well supported, however, *C. schleichi* as currently understood includes several separate species. The problem is presently under revision (Huemer in prep.).

[179] *Caryocolum marmorea* spp. *mediocorsa* agrees in DNA barcode with the nominotypical subspecies.

[180] *Caryocolum pullatella*. This species shows an extraordinary genetic variation across its holarctic range (Mutanen et al. 2012) and is in strong need of taxonomic re-assessment. In Europe two geographically separated DNA barcode clusters with separate BINs are present.

[181] *Caryocolum klosi*. A single DNA barcode from the French Pyrenees is highly divergent with a separate BIN (4.17% min. distance) and may represent a different species.

[182] *Caryocolum blandella*. *Lita signatella* was described from an unstated number of specimens from Kazan in Russia (“provincia Casanensi”) (Eversmann 1844). The short description is insufficient for identifying the species. The type series in the Zoological Institute in St. Petersburg is apparently mixed. During earlier visits Klaus Sattler (in litt.) and OK examined alleged syntypes of *L. signatella* incorporated under that name and which proved conspecific with *Carpatolechia proximella* (Hübner), and thus *L. signatella* was formally synonymized with that species in the Russian checklist (Ponomarenko 2008). However, only a single specimen of *L. signatella* was mentioned in an earlier work on the collection of Eversmann (Bremer 1870) and this specimen was recently designated as the lectotype (Sinev et al. 2017). It is conspecific with *Caryocolum blandella* (Douglas) which thereby became a junior synonym of *L. signatella*.

Whereas the name *Caryocolum blandella* has been universally in use for a widespread European species since Kloet and Hincks (1972), *Lita signatella* has to our knowledge not been used as a valid name since 1899, and it is not listed in the main catalogues of the Gelechiidae (Rebel 1901, Meyrick 1925, Gaede 1937). According to Articles 23.9.1 and 23.9.2 of the International Code of Zoological Nomenclature (ICZN 1999), we therefore declare the name *Caryocolum blandella* Douglas, 1852 to be a nomen protectum, and the name *Lita signatella* Eversmann, 1844, which has not been used as a valid name after 1899, to be a nomen oblitum. Supplementary material 1 lists 35 references by more than ten different authors that have used *C. blandella* (or its alternative spelling *C. blandellum*) in the last 50 years (ICZN article 23.9.1.2). The name is moreover used in several other published works and on numerous internet sites.

[183] *Caryocolum horoscopa*. Initially described as a species, this taxon was recently considered to be a subspecies of *Caryocolum blandella* (Huemer and Karsholt 2010). However, in addition to diagnostic morphology, DNA barcodes also clearly support a separate species status for this taxon, and we therefore reinstate *C. horoscopa* stat. rev.

[184] *Caryocolum fibigerium*. Huemer et al. (2014) had indicated likely taxonomical problems in this group highlighted by three DNA barcode clusters on the Iberian, Italian and Balkan peninsulas. These genetic splits are also supported by morphological traits and probably reflect three different species (Huemer in prep.).

[185] *Caryocolum junctella*. Two barcode clusters with separate BINs show no clear geographic separation.

[186] *Agonochaetia terrestrella*. Specimens from Switzerland and Romania cluster into a separate BIN, but are considered as conspecific (Huemer and Karsholt 2010).

[187] *Sattleria*. The sequence of species follows Huemer and Timossi (2014).

[188] *Sattleria melaleucella*. The species shares BINs with one cluster of the morphologically different *S. pyrenaica*, indicating occasional introgression.

[189] *Sattleria pyrenaica*. A genetically variable species with five different BINs, one shared with *Sattleria melaleucella*. The species requires taxonomic re-assessment.

[190] Litini. Ponomarenko (2005, 2008) showed that Teleiodini is a junior synonym of Litini, described as Litidae by Bruand d’Uzelle (1859).

[191] *Schneidereria pistaciella* Weber, 1957. The systematic placement of this genus and species follows Huemer and Karsholt (2001).

[192] *Teleiodes vulgella* / *T. italica* / *T. brevivalva*. These three species differ strongly in the male genitalia but share DNA barcodes.

[193] *Teleiodes saltuum* / *T. kaitilai*. Both species are closely related, mainly differing in the structures of the female genitalia. In DNA barcodes *T. saltuum* clusters into two BINs and *T. kaitilai* in a separate BIN.

[194] *Teleiodes luculella*. A genetically variable species, which clusters into three BINs. A thorough evaluation of this problem is necessary.

[195] *Teleiodes flavimaculella*. A genetically highly variable species, which clusters into three BINs. A re-evaluation of this problem is necessary.

[196] *Pseudotelphusa tessella*. Two weakly separated BINs (1.61% min. distance) without clear geographic separation are considered as intraspecific variation.

[197] *Teleiopsis diffinis* / *T. bagriotella* / *T. albifemorella* / *T. paulheberti* / *T. rosalbella*. These closely related species differ in morphology whereas barcodes give a more complex pattern. Genetic differences are generally weak with partial BIN sharing (i.e., *T. rosalbella* / *T. albifemorella*) and/or likely introgression in some taxa, while high intraspecific variation - with two BINs in three species - indicates possible further cryptic diversity.

[198] *Xenolechia*. Species in this genus share DNA barcodes but differ in morphology (Huemer and Karsholt 1999).

[199] *Altenia scriptella*. Two BINs without clear geographic separation are considered as intraspecific variation.

[200] *Exoteleia dodecella*. The taxonomy of dark specimens in this group, mainly observed in Central Europe, is disputed, though usually these are considered as infrasubspecific variation (Huemer and Karsholt 1999). We were able to sequence large series of specimens across Europe and discovered that DNA barcodes of normal and dark specimens are usually separated by a low but constant barcode gap of about 1%. These results, in combination with differences in adult morphology, clearly indicate presence of two separate species. Revisionary work is currently under preparation (Huemer et al. in prep.).

[201] *Parachronistis albiceps*. Genetically variable species, which clusters into four BINs without clear geographic separation.

[202] “*Telphusa*”. The placement of *cistiflorella* Constant, 1890 in the genus *Telphusa* follows Sattler (1985), who pointed out that this placement should be regarded as tentative. The DNA barcode of *T. cistiflorella* clusters among genera placed in the Gelechiini, and the male genitalia are overall similar to those of *Mirificarma*, although they have no filament.

## Supplementary materials

Supplementary material 1 Selected references that have used the name *Caryocolum blandella* in the last 50 years Data type: references https://binary.pensoft.net/file/392495This dataset is made available under the Open Database License (http://opendatacommons.org/licenses/odbl/1.0/). The Open Database License (ODbL) is a license agreement intended to allow users to freely share, modify, and use this Dataset while maintaining this same freedom for others, provided that the original source and author(s) are credited.Peter Huemer, Ole Karsholt
