# Supplementary material for: Commented checklist of European Gelechiidae (Lepidoptera)
Source: Zookeys. 2020 Mar 24;921:65–140. doi: 10.3897/zookeys.921.49197 (PMC7109147; doi:10.3897/zookeys.921.49197)
Supplement: Supplementary material 1 [file zookeys-921-065-s001.doc]

**Supplementary material 1**

Selected references that have used the name *Caryocolum blandella* in the last 50 years.

Aarvik L, Bengtsson BÅ, Elven H, Ivinskis P, Jürivete U, Karsholt O, Mutanen M, Savenkov N (2017) Nordic-Baltic Checklist of Lepidoptera. Norwegian Journal of Entomology, Supplement 3: 1–236*.*

Aarvik L, Bakke SA, Berg Y, Berggren K, Hansen LO, Myhr K, Svendsen S (1997) Contribution to the knowledge of the Norwegian Lepidoptera V. Fauna norvegica Serie B 44: 55–70.

Burmann K (1990) Beiträge zur Microlepidopteren-Fauna Tirols. XIV. *Caryocolum* Gregor & Povolný, 1954 (Insecta: Lepidoptera, Gelechiidae). Berichte des naturwissenschaftlich-medizinischen Vereins Innsbruck 77: 171–184.

Buszko J, Nowacki J (2000) The Lepidoptera of Poland. A Distributional Checklist. Polish Entomological Monographs 1: 1–178 pp.

Corley MFV (2015) Lepidoptera of continental Portugal. A fully revised list. M. Corley, Faringdon, UK, vi + 282 pp.

Elsner G, Huemer P, Tokár Z (1999) Die Palpenmotten (Lepidoptera, Gelechiidae) Mitteleuropas. Bestimmung – Verbreitung – Flugstandort – Lebensweise der Raupen. Verlag F. Slamka, Bratislava, 208 pp.

Emmet AM (ed.) (1988) A field guide to the smaller British Lepidoptera (edn 2). British Entomological & Natural History Society, London, 288 pp.

Emmet AM, Langmaid JR (2002) The moths and butterflies of Great Britain and Ireland. Vol. 4 (Part 2) Gelechiidae. Harley Books, Colchester, 277 pp., 63 text-figs, 6 colour pls.

Gaedike R, Heinicke W (eds) (1999) Verzeichnis der Schmetterlinge Deutschlands. Entomologische Nachrichten und Berichte, Beiheft 5, 216 pp.

Haslberger A, Segerer AH (2016) Systematische, revidierte und kommentierte Checkliste der Schmetterlinge Bayerns (Insecta: Lepidoptera). Mitteilungen der Münchner Entomologischen Gesellschaft 106, Suppl., 336 pp.

Huemer P (1988) A taxonomic revision of *Caryocolum* (Lepidoptera: Gelechiidae). Bulletin of the British Museum (Natural History) Entomology 57: 439–571.

Huemer P (1993) The British species of *Caryocolum* Gregor & Povolny. British Journal of Entomology and Natural History 6: 145–157, pl. 5.

Huemer P (2013) Die Schmetterlinge Österreichs (Lepidoptera). Systematische und faunistische Checkliste. Studiohefte 12, 304 pp.

Huemer P, Karsholt O (2010) Gelechiidae II (Gelechiinae: Gnorimoschemini). In*:* Huemer P, Karsholt O, Nuss M (eds): Microlepidoptra of Europe, vol. 6. Apollo Books, Stenstrup, 586 pp.

Huemer P, Karsholt O, Mutanen M (2014) DNA barcoding as a screening tool for cryptic diversity: an example from *Caryocolum*, with description of a new species (Lepidoptera, Gelechiidae). ZooKeys 404: 91–111. <https://doi.org/10.3897/zookeys.404.7234>

Huemer P, Tarmann G (1993) Die Schmetterlinge Österreichs. Systematisches Verzeichnis mit Verbreitungsangaben für die einzelnen Bundesländer. Veröffentlichungen des Museums Ferdinandeum, Beilageband 5: 1–224.

Huisman KJ, Koster JC, van Nieukerken EJ & Ulenberg SA (2001) Nieuwe en interessante Microlepidoptera uit Nederland in het jaar 1999 (Lepidoptera). Entomologische Berichten 61: 169–199.

Junnilainen J, Karsholt O, Nupponen K, Kaitila J-P, Nupponen T, Olschwang V (2010) The gelechiid fauna of the southern Ural Mountains, part II: list of recorded species with taxonomic notes (Lepidoptera: Gelechiidae). Zootaxa 2367: 1–68. DOI: [10.11646/zootaxa.2367.1.1](https://www.researchgate.net/deref/http%3A%2F%2Fdx.doi.org%2F10.11646%2Fzootaxa.2367.1.1?_sg%5B0%5D=hmpkLkXmha18_z84z8HVAzdFbaQCQKCxEoDsx0tEdOYfT7KggAhiAY_6cIKpa_0ekdbuJ0i-Y21BtJoFTgHkVVHTrA.gu-1Ld5k3S-ZaJLw9P0tkTXroJ6c5lI_bv72InjRhFuxdZfZFiCjVH8eE7DUG9mHC_MpsRkaGAJGzxVcqEYtOQ)

Karsholt O (1981) Northern European species of the genus *Caryocolum* Gregor & Povolny, 1954, feeding on *Cerastium* and *Stellaria*, with description of a new species (Lepidoptera: Gelechiidae). Entomologica scandinavica 12: 251–270.

Karsholt O (2004–2019) Gelechiidae. In: Karsholt O and van Nieukerken EJ (eds): Lepidoptera. Fauna Europaea [last update of Gelechiidae: version 2.4 January 2011]. <https://fauna-eu.org/>

Karsholt O, Mutanen M, Lee S, Kaila L (2013) A molecular analysis of the Gelechiidae (Lepidoptera, Gele­chioidea) with an interpretative grouping of its taxa. Systematic Entomology 38: 334–348.

Karsholt O, Nielsen PS (2013) Revised checklist of the Lepidoptera of Denmark. Lepidopterologisk Forening, København, 120 pp.

Karsholt O, Razowski J (eds) (1996) The Lepidoptera of Europe. A distributional checklist. Apollo Books, Stenstrup, 380 pp.

Kloet, GS & Hincks, WD (1972) A Check List of British Insects. 2^nd^ Ed, (Revised) Part 2: Lepidoptera. Handbooks for the identifications of British insects 11(2). Royal Entomological Society, London, viii + 153 pp.

Kozlov MV, Kullberg J, Zverev VE (2017) New records of Lepidoptera from the Arkhangelsk oblast of Russia. Entomologica Fennica 28: 169–182.

Kuchlein JH (1993) De kleine vlinders. Pudoc, Wageningen, 715 pp.

Laštůvka Z (ed.) (1998) Checklist of Lepidoptera of the Czech and Slovak Republica (Insecta, Lepidoptera). Konvoj, Brno, 118 pp.

Laštůvka Z, Liška J (2011) Annotated checklist of moths and butterflies of the Czech Republic (Insecta: Lepidoptera). Biocont Laboratory spol. s r.o., Brno, 146 pp.

Leraut PJA (1997) Liste systématique et synonymique des lépidoptères de France, Belgique et Corse (deuxième édition). Alexanor (Supplément): 1–526.

Lesar T, Habeler H, Arenberger E (2009) Prispevek k poznavanju metuljev (Lepidoptera) Slovenije II: nove vrste metuljckov (Microlepidoptera). Natura Sloveniae 11(2): 39–60.

Piskunov VI (1990) Gelechiidae. In: Medvedev GS (ed.) Keys to the Insects of the European part of the USSR, IV. Leningrad, Lepidoptera 2: 889–1024. [English translation].

Sobczyk T, Stöckel D, Graf F, Jornitz H, Karisch T, Wauer S (2018) Die Schmetterlingsfauna (Lepidoptera der Oberlausitz. Teil 5: Kleinschmetterlinge (Microlepidoptera) 1. Teil. Entomologische Nachrichten und Berichte, Beiheft 22, 439 pp.

Sterling P, Parsons M (2012) Field guide to the Micro moths of Great Britain and Ireland. British Wildlife Publishing, Gillingham, 416 pp.

SwissLepTeam (2010) Die Schmetterlinge (Lepidoptera) der Schweiz. Eine kommentierte, systematisch-faunistische Liste. Fauna Helvetica 25: 1–349.

Vives Moreno A (2014) Catálogo sistemático y sinonímico de los Lepidoptera de la Península Ibérica, de Ceuta, de Melilla y de las Islas Azores, Baleares, Canarias, Madeira y Salvajes (Insecta: Lepidoptera). Improitalia, Madrid, 1184 pp.
